# Recombinant LAB vector-based multicomponent vaccine against *Campylobacter jejuni* potentially promoting a healthier microbial balance in the poultry gut

**DOI:** 10.1186/s40168-026-02421-w

**Published:** 2026-05-07

**Authors:** Prakash Biswas, Sakil Ahmed, Samiran Mondal, Samson Oladokun, Ozan Gundogdu, Amirul Islam Mallick

**Affiliations:** 1 Department of Biological Sciences, Indian Institute of Science Education and Research Kolkata, Mohanpur, Nadia, West Bengal 741246 India; 2 Department of Veterinary Pathology, West Bengal University of Animal and Fishery Sciences, Kolkata, West Bengal 700037 India; 3 Department of Poultry Science, Texas A&M University, College Station, TX 77843 USA; 4 Department of Infection Biology, Faculty of Infectious & Tropical Diseases, London School of Hygiene and Tropical Medicine, London, WC1E 7HT UK

**Keywords:** *Campylobacter jejuni*, Chickens, *Lactococcus lactis*, Chitosan, Multicomponent vaccine, Gut health

## Abstract

**Background:**

Diarrheal diseases remain the second leading cause of preventable death globally, particularly among children under the age of 5 in developing countries, accounting for an estimated 2–3 million deaths annually. Among bacterial pathogens causing diarrheal illness, *Campylobacter jejuni* (*C. jejuni*) remains a major contributor, particularly in low- and middle-income countries (LMICs). As a common gut pathogen, *C. jejuni* expresses several secretory or surface-expressed colonization proteins (SECPs), namely haemolysin co-regulated protein (Hcp), valine glycine repeats G (VgrG), *Campylobacter* adhesion to fibronectin (CadF), fibronectin-like protein A (FlpA), and jejuni lipoprotein A (JlpA). Most of these proteins play pivotal roles in bacterial self-survival, host-cell adhesion, and invasion of avian and non-avian hosts. To minimize *C. jejuni* adhesion and subsequent colonization in the avian gut*,* we explored the potential of a multicomponent mucosal vaccine composed of CadF, Hcp, and JlpA protein of *C. jejuni.*

**Results:**

For this purpose, we bioengineered a food-grade Lactic Acid-producing Bacterium, *Lactococcus lactis* (*L*. *lactis*), to express three key immunogenic subunits of *C. jejuni*, CadF, Hcp, and JlpA. Utilizing this live vector-based multicomponent mucosal vaccine platform, we investigated the immunoprotective potential of these antigens in chickens. Since the particular strain of *L. lactis* is non-colonizing, we used chitosan, a natural mucoadhesive, biodegradable polymer, to microencapsulate the engineered bacteria and increase their gut retention time for optimal interaction with local immune cells*.* Our in vivo immunization study demonstrated that oral administration of this multicomponent vaccine formulation elicited a strong local antibody response (sIgA) (*p* < 0.0001) and upregulated key pro-inflammatory cytokines, leading to robust mucosal immune protection (~ 1.54 log_10_ reduction) against the cecal colonization of *C. jejuni*. Beyond targeting *C. jejuni*, we hypothesized that the vaccine may influence the overall gut microbiota, potentially promoting a healthier microbial balance in the poultry gut. To this end, gut metagenomic analysis of vaccinated birds revealed a marked reduction in the phylum Campylobacterota (~ 2-fold), accompanied by increased abundance of the phyla Bacteroidota, as part of a beneficial microbial community.

**Conclusions:**

Together, this study underscores the potential of a live vector-based, multicomponent mucosal vaccine as a promising, cost-effective strategy to reduce the cecal load of *C. jejuni*, potentially limiting the risk of foodborne transmission in poultry production systems.

**Graphical Abstract:**

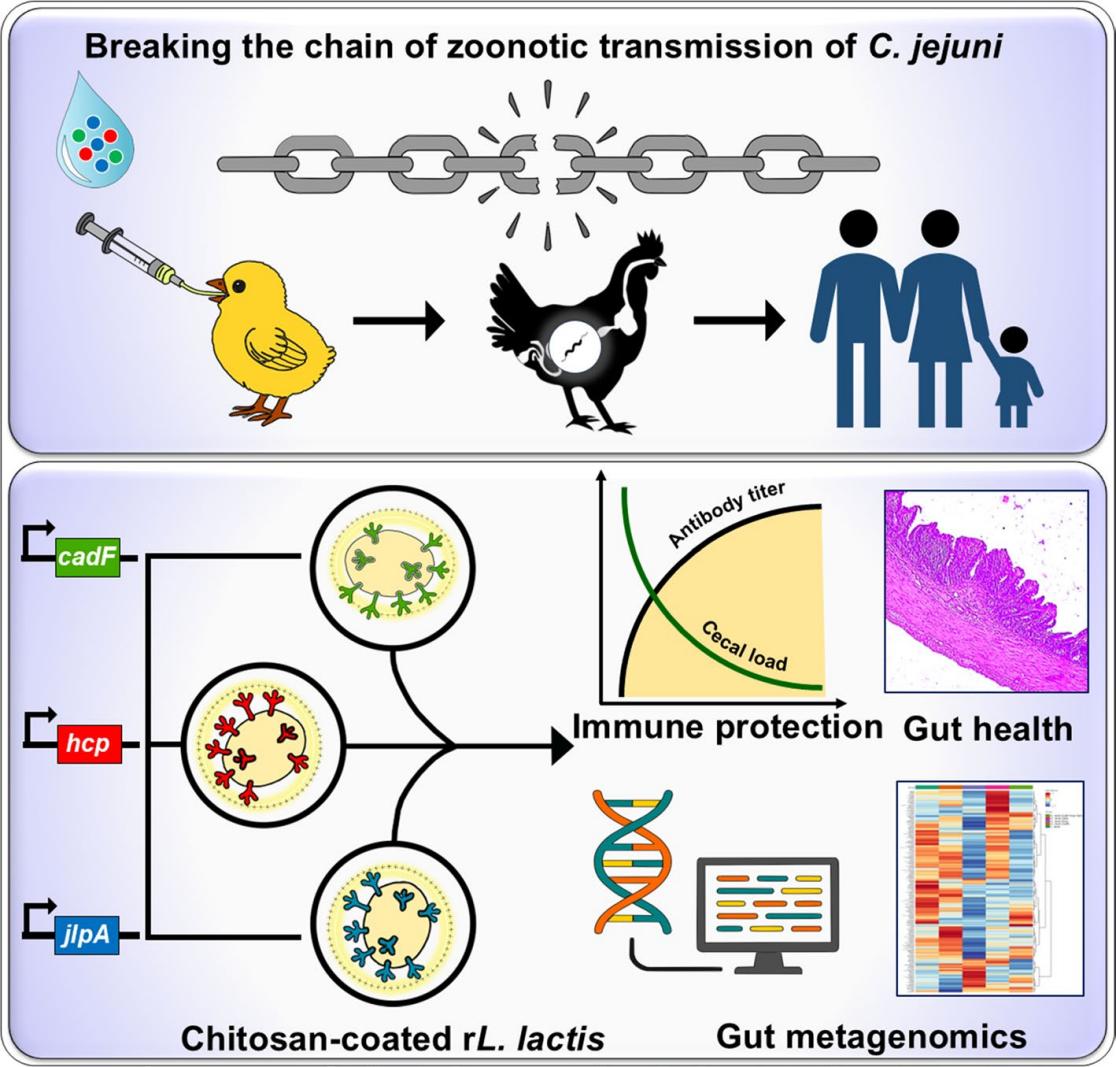

Video Abstract

**Supplementary Information:**

The online version contains supplementary material available at 10.1186/s40168-026-02421-w.

## Introduction

Diarrheal diseases are the second leading cause of preventable death globally, particularly affecting children under the age of 5 in developing countries, with an estimated 2–3 million deaths annually [1–3]. Bacterial diarrheal illnesses are primarily transmitted via the faeco-oral route and are commonly caused by *Escherichia coli*, *Yersinia enterocolitica*, Non-typhoidal *Salmonella* (NTS), *Shigella*, *Vibrio cholerae*, and *Campylobacter jejuni* (*C. jejuni*) [4–8]. Among these pathogens, *C. jejuni* is recognized as the leading bacterial cause of human diarrheal disease and is especially prevalent in low- and middle-income countries (LMICs) [9, 10]. Although *C. jejuni* infects a wide range of hosts, poultry, particularly broilers, are the primary reservoir and a significant source of human infection [11, 12]. Intestinal colonization of *C. jejuni* in poultry is typically asymptomatic; however, high bacterial loads can cause moderate-to-severe pathogenicity, disrupting epithelial integrity, promoting extraintestinal dissemination, and increasing the risk of carcass contamination during meat handling and processing [13]. Consequently, handling or consumption of contaminated poultry products remains the primary route of human campylobacteriosis, which accounts for ~ 95 million gastroenteritis cases globally each year, underscoring the urgent need for effective strategies to control *C. jejuni* colonization in poultry and limit transmission to humans [14].

Recently, we demonstrated the role of key secreted and surface-expressed colonization proteins (SECPs) of *Campylobacter jejuni*, such as Hcp and JlpA, in mediating host cell adhesion, cellular invasion, and subsequent pathogenesis in both avian and non-avian hosts [15, 16]. Furthermore, mucosal administration of either Hcp or JlpA modestly reduced *C. jejuni* cecal colonization in chicken and murine models [15, 16]. Given that cecal colonization by *C. jejuni* is a multifactorial process involving several putative virulence factors, we explored the efficacy of a multicomponent vaccine formulation comprising JlpA (lipoprotein that binds explicitly to Hsp90α), Hcp (a key effector protein of bacterial T6SS), and CadF (a critical adhesion protein that facilitates host cell binding via fibronectin) [15–17]. Moreover, we hypothesized that because the sequences of the selected target genes (*cadF*, *hcp*, and *jlpA*) are highly conserved across *C. jejuni* strains, their combined use may provide “broad-spectrum” immune protection against *C. jejuni* [18–20]*.*

Building on this proposition, we employed our previously established mucosal vaccine delivery platform using a food-grade Lactic Acid-producing Bacterium (LAB) to surface-express the CadF, Hcp, and JlpA proteins of *C. jejuni* [21]. The specific strain used for this study was a direct engineered derivative of *Lactococcus lactis* subsp. *cremoris* (MG1363), a Generally Recognized As Safe (GRAS) category LAB vector (NZ9000) [22]. Given that this strain of *L. lactis* is non-colonizing by nature, we used chitosan (CS), a natural, mucoadhesive, and biodegradable polymer, to microencapsulate recombinant *L. lactis* (r*L. lactis*) cells [23, 24]. This encapsulation aimed to enhance the stability and gut retention of *L. lactis* and promote optimal interaction with local immune cells, thereby improving the overall mucosal immunogenicity against the target protein [24]. Finally, the mixture of CS-coated r*L. lactis* in optimal numbers (3 × 10^9^ CFU) was orally administered (oral gavage) in 7-day-old *Campylobacteria*-free chicks (Rhode Island Red) for three consecutive weeks, followed by a challenge infection with a highly colonizing strain of *C. jejuni* (TGH 9011).

We demonstrated that oral administration of this multicomponent vaccine formulation elicited a strong local immune response, characterized by a marked increase in secretory IgA (sIgA) levels and the expression of key pro-inflammatory cytokines (such as IL-8, IL-1β, IL-17A). Histopathological analysis of the challenged birds immunized with the present vaccine further supported the positive impact of the combinatorial vaccine strategy using a LAB-based delivery platform on overall gut health. These findings align with the general health benefits of probiotics, which include the inhibition of colonizing pathogens through competitive adhesion to the epithelium and the production of antimicrobial substances (such as bacteriocins), especially by LAB vectors [25, 26].

Since *C. jejuni* exhibits little or no pathogenicity in the chicken gut, we were interested in assessing how the bioengineered LAB vector expressing heterologous proteins influences the gut microbiome [27]. We employed full-length 16S rRNA gene sequencing (~ 1500 bp) using long-read sequencing technology to achieve higher taxonomic resolution of microbial populations [28]. Our metagenomics data and the in vivo challenge experiment reveal an overall reduction in *Campylobacter* spp., accompanied by increased abundance of *Lactobacillus* spp. and other beneficial microbiota, compared to unvaccinated control birds. In particular, metagenomic data and histopathological analysis indicate improved gut health and a more favorable microbial composition, which is at least partly attributable to the intrinsic probiotic effects of the LAB vector used as a vaccine delivery platform. This vaccine strategy offers a promising alternative to traditional antibiotic use in poultry farming, aligning with global efforts to reduce antibiotic resistance. By targeting *C. jejuni* colonization and modulating the gut microbiota, the vaccine could enhance poultry health and food safety. Together, the present multicomponent vaccine against *C. jejuni* demonstrates significant benefits, including a marked reduction in *C. jejuni* colonization, highlighting the effectiveness of targeting multiple virulence factors for enhanced protective immunity.

## Materials and methods

### Bacterial strains, cell lines, and other reagents

#### Bacteria

The list of bacterial strains and plasmids used in this study is presented in Table S1 (Supplementary information). *Lactococcus lactis* subsp. *cremoris* NZ9000 cells and all recombinant *L. lactis* strains were routinely cultured at 30 ℃ in GM17 broth (HiMedia, India) without shaking. When required, chloramphenicol (SRL, India) was used at a final concentration of 20 µg/mL for recombinant strains. The recombinant *E. coli* strains (TOP10), harboring plasmids, were cultured in Luria–Bertani (LB) broth (Himedia) at 37 ℃ containing 20 µg/mL of chloramphenicol [16]. *C. jejuni* TGH 9011 was obtained through BEI Resources, NIAID, NIH: *Campylobacter jejuni* subsp. *jejuni*, Strain TGH 9011, NR-4082. *C. jejuni* was cultured at 37 ℃ in Muller-Hinton (MH) broth (Himedia) supplemented with CAT (Cefoperazone, Teicoplanin, Amphotericin B) selective supplement (Himedia) in a tri-gas incubator.

#### Cell lines

Nineteen-day-old embryonated eggs were used to isolate primary chicken embryo intestinal cells (CEICs) and cultured as described earlier. For CEICs, intestinal sections of 19-day-old embryos were aseptically collected and cut into small pieces in 1 × phosphate-buffered saline (PBS). After that, 0.25% trypsin–EDTA was added, and the tissue was stirred for 45 min at room temperature (RT). Cells were isolated from the suspension through a cell strainer (40 µm) and centrifuged at 500×*g *for 10 min at RT. Isolated primary CEICs were maintained in RPMI 1640 (Gibco, Invitrogen) medium supplemented with 10% fetal bovine serum (FBS), 100 μg/mL streptomycin, and 100 U/mL penicillin at 37 °C with 5% CO_2_ for 4–6 days until confluency reached 70–80% [29].

#### Chemicals and reagents

The reagents and chemicals used in this study were of the highest purity available. Chitosan was purchased from HiMedia Laboratory (India), and Sodium Tripolyphosphate (TPP) was procured from Loba Chemie (India).

### Generating recombinant *L. lactis* (r*L. lactis*) surface expressing CadF, Hcp, and JlpA protein of *C. jejuni*

**Fig. 1:**
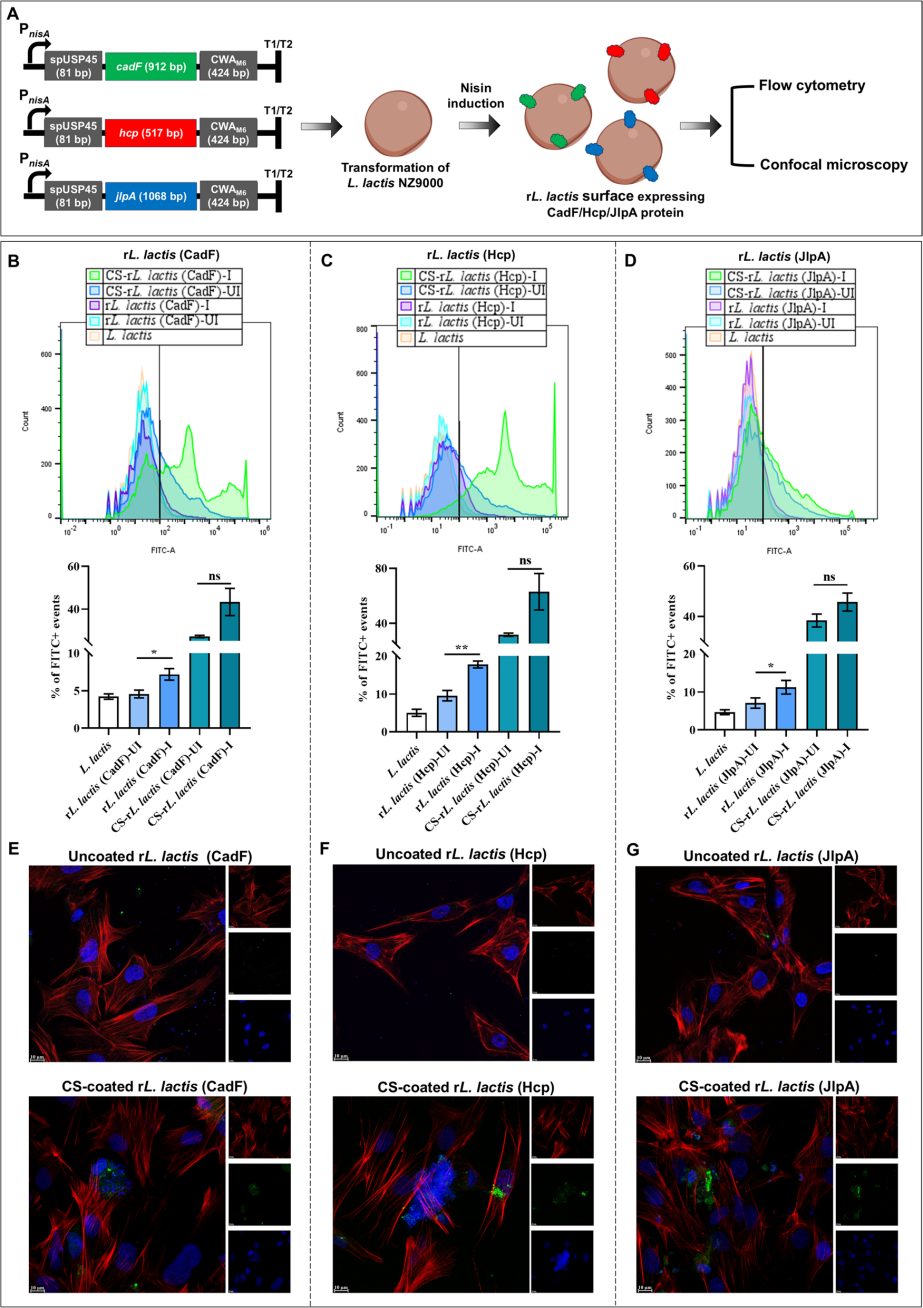
Nisin-induced CS-coated r*L. lactis* (CadF, Hcp, and JlpA) surface expression and adhesion to host cells. **A **Schematic representation of the cloning, expression, and validation of surface display of CadF, Hcp, and JlpA in r*L. lactis. ***B**–**D **Surface expression of CS-coated r*L. lactis* (CadF, Hcp, and JlpA) by flow cytometry. Histograms denote the FITC-A signal vs counts in each group, and the bar graph for % of FITC^+^ events among the groups. Data shows enhanced signals in the CS-coated and uncoated induced (I) groups compared to the CS-coated and uncoated uninduced (UI) groups. Each bar represents the mean % of FITC^+^ events ± SE from two independent experiments (*n* = 5). Asterisks indicate a statistically significant difference (**p < 0.01) compared to the control group. **E**–**G **In vitro adhesion assay of uncoated and CS-coated r*L. lactis* (CadF, Hcp, JlpA) in primary chicken embryo intestinal cells (CEICs). The cells were incubated with induced uncoated and CS-coated r*L. lactis* for 4 h at 37 °C with 5% CO_2_, at a ratio of cell vs bacteria 1:1000. Representative CLSM images of cells incubated with CS-coated r*L. lactis* cells showed enhanced cell adhesion compared to uncoated bacteria. Scale bar: 10 μm

For generating r*L. lactis* surface-expressing *C. jejuni* JlpA (1068 bp) and Hcp (517 bp) antigens without the signal peptide, we used our previous constructs with the pNZ8048 backbone. Briefly, the DNA sequences corresponding to *jlpA* and *hcp* coding sequences were PCR-amplified from the pQE30-JlpA and pQE30-Hcp plasmids. The amplified sequences were double-digested with *Sph*I and *Nhe*I and cloned into the pNZ8048 backbone, in frame with the C-terminal cell wall anchoring motif (CWA_M6_; 424 bp) of the *Streptococcus pyogenes* M6 protein [15, 16]. For the CadF (912 bp), the sequence without the signal peptide was amplified by PCR from the genomic DNA of *C. jejuni* (BCH71) (Table S4, Supplementary Information). The restriction-digested amplicon was cloned between downstream of a signal peptide (_SP_) of *L. lactis* protease USP45 (_SP_USP45; 81 bp, includes 27 residues of the USP45 leader peptide and a cleavage site for signal peptidase) and upstream of the CWA_M6_ motif using *Sph*I and *Nhe*I restriction enzymes as per the published method [15, 16]. The constructs were then transformed into *E. coli* TOP10 and incubated in LB agar plates supplemented with chloramphenicol (20 µg/mL) for screening positive transformations. Recombinant plasmids harbouring JlpA, Hcp, and CadF were isolated from *E. coli* and electrotransformed into *L. lactis* NZ9000 cells using GenePulser (BioRad, USA) (2000 V, 25 µF, 200 Ω) as per the method described previously [30]. Finally, the expression of the target protein was induced by the optimal concentration of Nisin (Sigma, USA) (15 ng/mL) [31] (Fig. 1A) (Fig. S1, Supplementary information).

### Expression and purification of recombinant Hcp, JlpA, and CadF proteins in *E. coli*

To prepare stocks of recombinant purified proteins for use in different immunoassays, the genes encoding Hcp and JlpA were cloned into *E. coli* expression vectors (pQE30, Qiagen) to enable the expression and purification of the target proteins. For CadF, the gene was amplified from *C. jejuni* TGH9011 genomic DNA, cloned into the *E. coli* expression vector pHisTEV (Bio Bharati Life Sciences, India), and the recombinant protein was expressed in competent *E. coli* cells. For JlpA and Hcp, we used our previously constructed expression vectors, which were already optimized for recombinant protein production in *E. coli* [16, 32]. In brief, the transformed *E. coli* cells were grown in LB medium containing the appropriate antibiotic and induced with isopropyl β-D-1-thiogalactopyranoside (IPTG) for recombinant protein expression. Following induction, the cells were harvested and lysed, and the recombinant proteins were purified using Ni–NTA affinity chromatography under appropriate conditions. The size and purity of the purified proteins were determined by SDS-PAGE analysis.

### Chitosan (CS) coating of *L. lactis* and r*L. lactis* surface expressing CadF, Hcp, and JlpA protein

To enhance the stability and mucosal adherence of r*L. lactis* expressing CadF, Hcp, and JlpA proteins, chitosan (CS) coating was performed. The coating was performed via ionic gelation with sodium tripolyphosphate (TPP) as described below. Approximately 4 h post-induction with nisin, *L. lactis,* and r*L. lactis* cells (~ 3 × 10^9^ CFU) were centrifuged at 2400×*g *for 5 min to collect the bacterial pellet. Then, the bacterial pellet was resuspended in 1 × PBS (700 µL), added to 0.05% (w/v) CS solution (3.3 mL), and stirred for 30 min at RT. After incubation, 1 mL of 0.1% (w/v) sodium tripolyphosphate (TPP) solution was added dropwise, and the mixture was further incubated for 1 h under stirring at RT. Then, the solution was pelleted and washed thrice with 1 × PBS to remove residual unpolymerized CS and TPP using centrifugation at 2400×*g *for 5 min. Finally, the coated r*L. lactis* cells were resuspended in 1 × PBS for further assessment [29].

### Determining the accessibility of the recombinant protein expressed by CS-coated r*L. lactis*

To evaluate the accessibility of surface-expressed antigens and the effect of chitosan (CS) coating on protein accessibility, five bacterial groups were prepared for each antigen and replicate. Approximately 3 × 10^9^ CFU of CS-coated or uncoated recombinant *Lactococcus lactis* (r*L. lactis*) surface-expressing Hcp, JlpA, and CadF (either nisin-induced or uninduced) were processed for flow cytometry as described previously [16]. The five sets of bacteria included: wild-type *L. lactis* (*L. lactis*), uninduced recombinant *L. lactis* (r*L. lactis*-UI), nisin-induced recombinant *L. lactis* (r*L. lactis*-I), CS-coated uninduced recombinant *L. lactis* (CS-r*L. lactis*-UI), and CS-coated induced recombinant *L. lactis* (CS-r*L. lactis*-I). Wild-type *L. lactis* served as the negative control. All samples were processed similarly for antibody labeling. Briefly, bacterial cells were first fixed with pre-chilled 4% paraformaldehyde (PFA) and subsequently blocked with 3% bovine serum albumin (BSA) in PBS to prevent non-specific binding. Following blocking, cells were washed and probed with anti-CadF/Hcp/JlpA antibodies raised in mice/rabbit (1:100 dilution) overnight at 4 ℃. After incubation, cells were washed with PBS and incubated with FITC-conjugated goat anti-mice/rabbit IgG antibody (H + L) (1:500 dilution) (Thermo Fisher Scientific, USA) for 2 h at room temperature. Finally, the cells were washed and subjected to flow cytometric analysis in BD LSR Fortessa using the FITC filter (Excitation/Emission at 488/535 nm) [15, 16, 29].

### In vitro cell adhesion of CS-coated r*L. lactis*

The mucoadhesive property of chitosan-coated r*L. lactis* expressing rCadF, rHcp, and rJlpA proteins to primary chicken embryo intestinal cells (CEICs) was assessed using confocal microscopy. Briefly, nisin (15 ng/mL) induced uncoated or CS-coated r*L. lactis* cells were incubated with CEICs at a ratio of 1:1000 (CEICs: bacteria) for 4 h at 37 ℃. Then, cells were washed with PBS and fixed with 4% PFA. After 15 min, cells were thoroughly washed and blocked using 3% BSA for 1 h at RT, followed by probing with an antigen-specific primary antibody raised in mice/rabbit (1:100 dilution) for 2 h at RT. After three washes, cells were stained with FITC-conjugated goat anti-mice/rabbit IgG (H + L) secondary antibody (1:500 dilution) (Thermo Fisher Scientific, USA). Further, the cells were stained with phalloidin 647 and 4′,6-diamidino-2-phenylindole (DAPI) and mounted onto a glass slide with Vectashield mounting media (Vector Laboratories, USA) for confocal microscopy, and images were captured (405 nm laser for DAPI; 488 nm laser for FITC; 638 nm laser for Phalloidin 647) [29].

### In vivo immunogenicity of mucosally administered r*L. lactis* in chickens

#### Immunogen preparation and experimental groups

Recombinant *L. lactis* displaying rCadF, rHcp, and rJlpA proteins were expressed using the optimal nisin concentration (15 ng/mL). To coat with chitosan-TPP, the induced cells were pelleted, washed with sterile endotoxin-free PBS, and resuspended in PBS. To assess the in vivo immunogenicity of mucosally (intra-gastric) administered *L. lactis* cells expressing rCadF, rHcp, and rJlpA proteins, a total of 180 Rhode Island Red (RIR) chicks were purchased from ARD RKVY Haringhata Farm (India). The experimental birds were maintained in a deep litter system throughout the trial and fed an ad libitum antibiotic-free corn-soybean-based mash diet. The detailed composition of the substrate used in this study is provided in Table S2 (Supplementary information). On day 7, the chicks were randomly assigned to 7 experimental groups (24 birds per group), and 6 additional birds were assigned to each of the baseline control and unimmunized-unchallenged (UU) groups. Chicks from different groups received the following treatments in 100 µL of PBS. Control Group (PBS): 100 µL PBS/bird; Group CS: 100 µL CS-TPP/bird; Group *L. lactis*: 3 × 10^9^ CFU *L. lactis* NZ9000/bird; Group r*L. lactis* (CadF): 1 × 10^9^ CFU r*L. lactis*-CadF with 2 × 10^9^ CFU NZ9000/bird; Group r*L. lactis* (JlpA): 1 × 10^9^ CFU r*L. lactis*-JlpA with 2 × 10^9^ CFU NZ9000/bird; Group r*L. lactis* (Hcp): 1 × 10^9^ CFU r*L. lactis*-Hcp with 2 × 10^9^ CFU NZ9000/bird; Group r*L. lactis* (CadF + Hcp + JlpA): 1 × 10^9^ CFU r*L. lactis*-CadF + 1 × 10^9^ CFU r*L. lactis*-Hcp + 1 × 10^9^ CFU r*L. lactis*-JlpA/bird.

#### Sample collection

At day 5 post-last feeding (day 28), half of the birds from each group were sacrificed to collect the Bursa of Fabricius (BOF), cecal tonsils, spleen, blood, feces, and intestinal lavages. In contrast, the remaining (*n* = 12) birds from each group were challenged with 1 × 10^8^ CFU of *C. jejuni* TGH 9011 (ATCC 43431). All challenged birds were euthanized on the seventh post-challenge day (day 35). Birds were euthanised by CO_2_ asphyxiation (30%) followed by cervical dislocation as per the guidelines approaved by the Committee for the Control and Supervision of Experiments on Animals (CCSEA), Ministry of Fisheries, Animal Husbandry and Dairying Department of Animal Husbandry and Dairying, Govt. of India (Source: Manual for CCSEA Guidelines for Polutry/Birds Facility, 2020).

For every bird in each group, samples of the BOF and spleen weighing around 100 mg were collected and stored in Trizol at − 80 ℃. Throughout the experiment, weekly blood and fecal samples were collected from individual birds in each group. Fecal pellets were processed using the previously mentioned procedure, while serum samples were aliquoted and kept at – 20 ℃ until further use [33]. Intestinal lavages were harvested following a published protocol standardized in our lab and stored at − 20 ℃ [16].

### Assessing local antibody responses in immunized chickens

#### Secretory IgA (sIgA) titer in intestinal lavages and fecal pellets

To evaluate the sIgA level against whole cell lysate (WCL) of *C. jejuni* TGH 9011 and recombinant proteins individually (CadF, Hcp, and JlpA) in clarified gastric lavage or fecal samples, indirect ELISA was performed using half-diluted samples (as starting dilution). Briefly, 1 µg/well of *C. jejuni* WCL and 100 ng/well of recombinant proteins (CadF, Hcp, and JlpA) were used to coat the ELISA plates overnight at 4 °C. The next day, plates were washed with PBST (0.1% Tween 20) and blocked using 5% BSA for 1 h. After thoroughly washing, intestinal lavages or fecal soup samples were added and incubated at RT for 2 h. Plates were then washed with PBST and probed with goat anti-chicken IgA HRP-conjugated as the secondary antibody (1:3000 dilutions; Bethyl Laboratories) at RT for 1 h. Following washes with PBST, 3,3′,5,5′-Tetramethylbenzidine (TMB) substrate was added to each well, and the reaction was stopped with 1 M H_2_SO_4_ after 5 min of incubation. Finally, the absorbance was read using a microplate reader (BioTek, Epoch2) at 450 nm [16].

### Assessment of cellular responses in immunized birds

#### Isolation of splenocytes

The spleens of five experimental birds (*n* = 5) per group were collected, and a single-cell suspension was prepared following the method described in previous studies [15, 34]. In brief, the spleens were minced in a sterile petri dish containing RPMI 1640 (Gibco, USA) medium using a disposable syringe plunger. The cell suspension was aspirated and passed through a 40 µm cell strainer (Corning, Merck). The resultant filtrate, comprising the single-cell suspension, was placed onto pre-warmed Histopaque-1077 solution (Sigma) in a 1:1 ratio and centrifuged at 500×*g *for 20 min at room temperature. The splenocyte interface was carefully harvested, washed, and resuspended in a complete growth medium (RPMI 1640).

#### In vitro nitric oxide (NO) production

To assess nitric oxide (NO) production by antigen-primed splenocytes from experimental birds, a standard Griess assay was performed according to the manufacturer’s instructions (Sigma). Briefly, a single-cell suspension of splenocytes was seeded in 24-well tissue culture plates for three hours at a density of 1.5 × 10^5^ in phenol red-free complete RPMI 1640 growth media (Gibco, USA). Then, splenocytes were challenged with *C. jejuni* WCL (1 μg/mL). Following 48 h of incubation, 100 μL of culture supernatant was collected from each well and incubated with an equal volume of Griess reagent at room temperature for 15 min. The absorbance for each well was measured at 540 nm using a microplate reader (Biotek, Epoch2) [35, 36]. The concentration of nitrite was determined in comparison with a standard curve generated using sodium nitrite (NaNO_2_) (Sigma) (Fig. S2, Supplementary information).

#### In vitro splenocyte proliferation

To evaluate cellular immune responses elicited by immunization, the splenocyte proliferation assay was performed using the Click-iT Plus EdU Alexa Fluor 488 Flow Cytometry Assay Kit (Invitrogen) according to the manufacturer’s protocol. Briefly, 3 × 10^5^ cells in RPMI 1640 medium were seeded into each well of a 12-well tissue culture plate. After 3 h, the cells were stimulated with 10 µg/mL of *C. jejuni* TGH 9011 whole-cell lysate (WCL). Unstimulated splenocytes and splenocytes stimulated with 10 µg/mL of concanavalin A (Con A) were used as controls. After 24 h of stimulation, 10 µM of 5-Ethynyl-2′-deoxyuridine (EDU) was added to the wells and incubated for an additional 24 h. The cells were then washed with PBS containing 0.2% BSA and fixed for 15 min on ice. Following fixation, the cells were rewashed and permeabilized for 15 min on ice. The cells were subsequently incubated on ice with EDU buffer additive and reaction cocktail for 30 min. After incubation, the cells were washed and analyzed by a BD LSRFortessa flow cytometer (BD Biosciences) [37]. The splenic lymphocyte proliferation index (Stimulation Index; SI) for each experimental group was calculated using the following formula: Stimulation Index (SI) = Mean fluorescence of stimulated cells/Mean fluorescence of unstimulated cells [38].

### Analysis of B and T cell subsets in cecal tonsils and Bursa of Fabricius (BOF)

#### Isolation of mononuclear cells from intestinal tissue

Whole cecal tonsils and a portion of the Bursa of Fabricius (BOF) were collected from chickens and kept on ice in PBS containing penicillin (10 U/mL) and streptomycin (10 µg/mL). Each tissue was then sectioned into smaller pieces and washed three times with PBS. Tissue samples were then digested with collagenase type I (4 mg/mL at 37 °C for 20 min; Merck) in PBS containing penicillin (100 U/mL) and streptomycin (100 µg/mL). The resulting tissue digests were filtered through 40-µm cell strainers (Corning, Merck), using the rubber end of a 10 mL syringe plunger to crush the tissue. The cell suspensions from the cecal tonsils and BOF were layered onto Histopaque-1077 (Sigma) in a 1:1 ratio, followed by density-gradient centrifugation at 500×*g *for 20 min to isolate the mononuclear cells. The aspirated buffy coats were washed by centrifugation at 400×*g *for 5 min in RPMI 1640 medium containing penicillin and streptomycin. The mononuclear cells were then resuspended in complete RPMI cell culture medium containing RPMI 1640, 10% FBS (Gibco), penicillin (100 U/mL), and streptomycin (100 µg/mL). Cell count and viability were assessed using a hemocytometer and the trypan blue exclusion method. The mononuclear cells were resuspended in complete RPMI medium at a concentration of 1 × 10^6^ cells/mL and kept on ice [23].

#### Flow cytometric analysis of B and T cell subsets

To evaluate the distribution of B and T cell subsets following immunization, mononuclear cells from cecal tonsils and BOF (1 × 10^6^ cells) were resuspended in FACS staining buffer (PBS containing 1% BSA) and subjected to staining with specific antibody panels. Cells were labeled with mouse anti-Bu1-FITC and mouse anti-IgA-PE to characterize B cells. For T cell identification, the cells were stained with mouse anti-CD3ζ-APC, mouse anti-CD4-FITC, and mouse anti-γδTCR-PE. The cells were incubated with the respective antibodies for 15 min at 4 °C in FACS staining buffer. After the staining procedure, cells were washed at 400×*g* for 5 min with FACS staining buffer and then incubated for an additional 10 min at 4 °C with 7-AAD (Southern Biotech, Canada). Following the incubation, cells were fixed with 4% paraformaldehyde (PFA) at 4 °C. After fixation, cells were washed and stored at 4 °C in FACS staining buffer [23]. Data were acquired on a BD LSRFortessa flow cytometer (BD Biosciences) and collected 5 × 10^4^ events. Flow cytometry data were subsequently analyzed using FlowJo V10 software. A detailed list of the antibodies used is provided in Table S3 (Supplementary information).

### Assessing cytokine gene expression in spleen and BOF tissues by quantitative real-time PCR (qRT-PCR)

To evaluate transcriptional expression of pro-inflammatory cytokines and immune-related genes following immunization, qRT-PCR was performed using RNA isolated from spleen and BOF tissues as described below. According to the manufacturer’s instructions, total RNA was isolated from 50 mg of spleen and BOF tissues using Trizol reagent (Invitrogen, USA). RNA was treated with DNase I to remove any contaminating DNA (DNase I, RNase-free, Thermo Scientific). Then, cDNA synthesis was performed for each sample using the iScript cDNA Synthesis Kit (Bio-Rad). To assess the expression of cytokine genes and transcription factors, qRT-PCR was performed using specific primers for chicken IFN-γ, IL-8, IL-1β, IL-17A, TNF-α, NF-κB, and iNOS, with chicken β-actin as the internal control. Primer details are provided in Table S4 (Supplementary information).

### In vitro functionality of sIgA against *C. jejuni* adherence to host cells

To evaluate the ability of intestinal sIgA to inhibit *C. jejuni* adherence and invasion, an in vitro protection assay was performed [34]. Briefly, 1.5 × 10^4^ *C. jejuni* cells were incubated with raw intestinal lavages for 3 h at 37 °C. After this incubation, chicken CEICs were incubated with the treated *C. jejuni* at an MOI of 1:100 for 3 h at 37 °C under 5% CO_2_. After incubation, the cells were washed and lysed with 1% Triton X-100. The lysates were serially diluted and plated onto *Campylobacter*-selective agar, which was then incubated overnight at 37 °C under microaerobic conditions in a tri-gas incubator (Thermo Fisher Scientific). Colonies that formed on the plates were counted for each experimental group.

### In vivo efficacy of mucosally administered r*L. lactis* against cecal colonization of *C. jejuni*

To evaluate the protective efficacy of r*L. lactis*, birds from different experimental groups were challenged orally with 1 × 10^8^ CFU of *C. jejuni* TGH 9011 (in 100 µL PBS) via oral gavage on day 5, following the last feeding. Seven days post-challenge, all birds were euthanized, and each bird’s cecal tissue and contents were collected.

#### Cecal load of *C. jejuni*

To determine the cecal load of *C. jejuni*, approximately 200 mg of cecal content from each bird was collected and dissolved in MH broth. Then, cecal contents were serially diluted in MH broth, plated onto *Campylobacter* selective agar supplemented with cefoperazone, amphotericin B, and teicoplanin (CAT) (Himedia), and incubated at 37 ℃ in a tri-gas incubator. The number of bacterial colonies on the plate was counted using a colony counter (Scan 500, Interscience, France) and plotted for each group.

#### Histopathological changes in cecal tissues

To evaluate the pathological alterations and mucosal tissue damage in the cecum following *C. jejuni* infection, histopathological examination of cecal tissues was performed as described below. On day 7 post-infection, cecal tissue from experimental birds was collected and processed for histological analysis in accordance with previously published protocols [39, 40]. Briefly, 0.5 cm sections of cecal tissue were initially fixed in 10% formalin, followed by washing under running tap water. The tissue was then dehydrated through a graded series of acetone (70%, 90%, and 100%). After dehydration, the tissues were cleared with two changes of benzene and subsequently embedded in molten paraffin (62 °C) through three 1 h changes. After paraffin block sectioning, hematoxylin and eosin (H&E) staining was performed to prepare the slides.

### Effect of oral administration of r*L. lactis* in the gut microbial population

#### Full-length 16S rRNA gene sequencing of the cecal microbial community

To evaluate the impact of oral administration of r*L. lactis* on the gut microbial community, full-length 16S rRNA gene sequencing of the cecal microbiota was performed using the Oxford Nanopore sequencing platform as described below. Total genomic DNA was extracted from 250 mg of caecal content from each bird (*n* = 4 per treatment group) with the PowerFecal Pro DNA Kit (QIAGEN, Germany). For microbial community analysis, 30 ng high-quality genomic DNA was used as an input for the Nanopore 16S Barcoding Kit 24 V14 (SQK-16S114.24), which amplified the 16S rRNA (V1-V9 regions) gene by PCR using specific barcodes and LongAmp Hot Start Taq 2X Master Mix (NEB, M0533) for each sample following the manufacturer’s instructions. A total of 36 samples were sequenced (4 samples/group, 9 groups), and the amplicons were quantified using Qubit dsDNA HS Assay Kit (Invitrogen, Q32851), pooled in equimolar concentration, and purified with AMPure XP beads (Beckman Coulter, USA). Fifty femtomoles of the barcoded library was ligated with Nanopore adaptors supplied in the 16S kit and sequenced on the FLO-MIN114 flow cell using the MinION Mk1B sequencer (Oxford Nanopore Technologies, UK) [28, 41].

#### Bioinformatics analysis of metagenomic data

FASTQ files from Nanopore sequencing were basecalled simultaneously during sequencing using the High Accuracy model v4.3.0 (400 bps, minimum Q score of 9) with MinKNOW (Oxford Nanopore Technologies, UK). The barcoded samples were analyzed using the wf-metagenomics workflow with EPI2ME v5.3.1 (Oxford Nanopore Technologies, UK). The sequences were referenced with the SILVA_138_1 database in the workflow. Then, a report of operational taxonomic units (OTUs) across all samples was generated.

Visualization and statistical analyses were conducted in MicrobiomeAnalyst. Alpha diversity was assessed using the Chao1, Shannon, Simpson, and Fisher diversity index, and statistical significance was determined using the Welch *t*-test/ANOVA and post hoc pairwise comparison. Beta diversity was visualized by principal coordinates analysis (PCoA) based on the Bray–Curtis index and PERMANOVA. Differential microbial abundance was evaluated using Linear Discriminant Analysis Effect Size (LEfSe), which applies the Kruskal–Wallis rank-sum test to identify significantly different features across classes, followed by linear discriminant analysis to estimate effect sizes (LDA score > 2, *p* < 0.05). Differentially abundant taxa were visualized using a dot plot. The Multiple Linear Regression with covariate adjustment was used for multi-factor analysis at the phylum level between groups, employing the Multivariable Association with Linear Models 2 (MaAsLin2) method with a Zero-Inflated Negative Binomial Model (ZINB) at an adjusted *p*-value cutoff of 0.05 [42, 43].

### Statistical analysis

Graphical presentations and data analysis were performed using GraphPad Prism statistical software (Version 8.0.1). Outliers were identified using the robust regression and outlier removal (ROUT) method and excluded from subsequent statistical analysis. The Shapiro–Wilk test was applied to assess the normality of the data. The Student’s t-test (two-tailed, unpaired) or the non-parametric Mann–Whitney *U *test was used to compare differences between experimental groups, depending on data distribution. Statistical significance was defined as *p < 0.05, **p < 0.01, ***p < 0.001 and ****p < 0.0001.

## Results

### Nisin-induced uncoated or CS-coated r*L. lactis* showed stable surface expression of CadF, Hcp, and JlpA proteins

To assess the accessibility of surface-expressed rCadF, rHcp, and rJlpA upon coating them with chitosan, induced r*L. lactis* cells were probed with polyclonal primary antibodies, followed by FITC-conjugated goat anti-mice/rabbit IgG (H + L) secondary antibody, and analyzed by flow cytometry. A distinct shift in fluorescence intensity was observed in the gated population of uncoated and CS-coated induced r*L. lactis* cells for all three recombinant proteins (CadF: *p* = 0.013, *p* = 0.003) (Hcp: *p* < 0.0001, *p* = 0.011) (JlpA: *p* = 0.012, *p* = 0.0002) compared to WT *L. lactis* NZ9000 (empty vector) cells (Fig. 1B–D).

### The CS-coating of r*L. lactis* enhances bacterial adhesion to host cells

We assessed the CS-coated r*L. lactis* cell adhesive performance to investigate whether electrostatic interactions between positively charged CS-coated bacteria enhance interactions with host cells. Confocal microscopy images showed that CS-coated induced r*L. lactis* expressing CadF/Hcp/JlpA had higher fluorescence signals and adhesive properties to primary CEICs than uncoated induced bacterial cells (Fig. 1E, F, and G) and (Fig. S3, Supplementary information).

### Oral administration of CS-coated r*L. lactis* induces significant local immune responses in chickens

#### Induction of local IgA (sIgA) responses

**Fig. 2:**
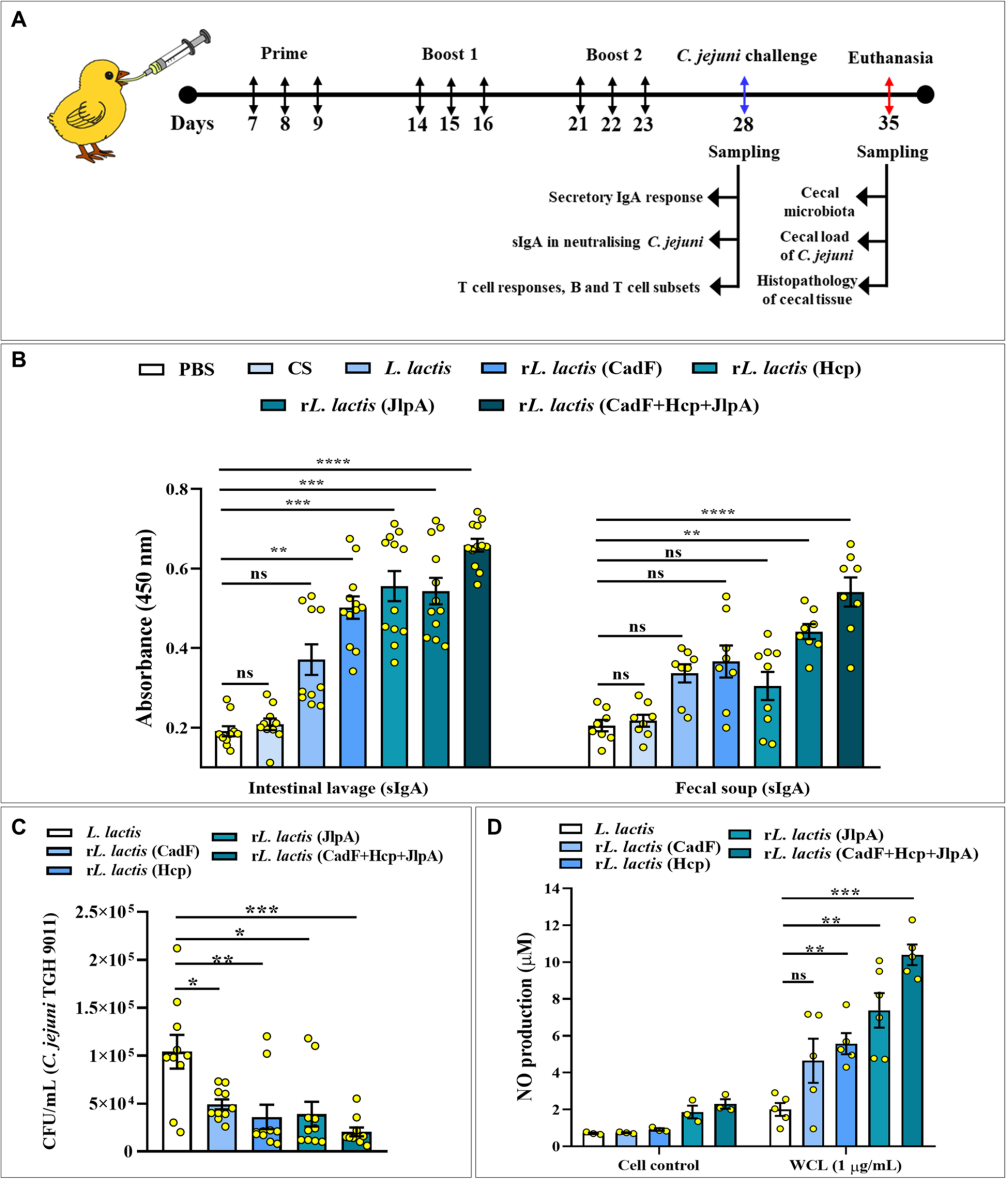
Schedule for oral administration of r*L. lactis* and assessment of antibody responses. **A **Schematic of the chicken immunization and sampling. Chickens were orally administered (oral gavage) with r*L. lactis* as per the indicated time points for 3 weeks for two independent experiments (*n* = 2). At day 5 post-last feeding (day 28), half of the birds were sacrificed to collect intestinal lavages, feces, blood, and tissue samples, while the remaining birds were challenged with 1 × 10^8^ CFU of *C. jejuni* (day 28). Challenged birds were sacrificed on day 35 to collect cecal tissue and its content.** B **Comparative analysis of mucosal (sIgA) antibody level in intestinal lavage, fecal soups collected from each bird belonging to different treatment groups. Data show a significant increase in sIgA levels in lavage and fecal soup from birds administered r*L. lactis* expressing CadF + Hcp + JlpA compared to the control group of birds (received PBS). Each bar represents the mean absorbance (A_450_) ± SE of 12 birds from two independent experiments. Outliers were detected and excluded using the robust regression and outlier removal (ROUT) method (*Q* = 1%). Asterisks indicate a statistically significant difference (***p < 0.001) compared to the control group (PBS).** C** In vitro neutralization of *C. jejuni* adhesion and invasion of chicken primary CEICs by secretory sIgA present in the lavage sample of birds belonging to the different experimental groups, showing marked reduction in *C. jejuni* adhesion and invasion exerted by r*L. lactis* (CadF + Hcp + JlpA) administered group of birds (**p < 0.01; immunized vs. control). Data represent mean CFU/mL ± SE (*n* = 10) from two independent experiments.** D** High-level nitric oxide (NO) production by the splenocytes collected from the birds administered with r*L. lactis* (CadF + Hcp + JlpA) when primed with the whole cell lysate of *C. jejuni*. Data represent mean NO production ± SE (*n* = 6). Outliers were detected and excluded using the ROUT method (*Q* = 1%)

To evaluate the ability of mucosal delivery of r*L. lactis* expressing CadF, JlpA, and Hcp to induce an antigen-specific local antibody response, intestinal lavages and fecal samples were collected on day 5 post-last immunization and analyzed for sIgA (Fig. 2A). Following intra-gastric administration of CS-coated r*L. lactis* cells over 3 weeks (3 consecutive days/week), a significant increase in local antibody level (secretory IgA) was observed in the intestinal lavage (*p* < 0.0001) and fecal soup (*p* < 0.0001) of the birds that received the combination of r*L. lactis* expressing all three proteins compared to the control group (PBS) (Fig. 2B). However, no significant changes (*p* > 0.999) in serum IgY response were observed among the groups (data not shown) [16].

Further, to determine the antigen-specific local antibody response against the individual recombinant proteins, CadF, Hcp, and JlpA were purified and analysed by SDS-PAGE. The gel images showed distinct protein bands at expected molecular weights corresponding to CadF (~ 20.4 kDa), Hcp (~ 20 kDa), and JlpA (~ 42 kDa) (Fig. S4, Supplementary information). ELISA plates were coated individually with purified recombinant CadF, Hcp, and JlpA proteins (100 ng/well), and sIgA levels were measured in intestinal lavage and fecal soup samples. When compared with the *L. lactis* group, birds immunized with r*L. lactis* strains expressing the respective antigens exhibited elevated sIgA responses against the coated proteins. In particular, immunization with r*L. lactis* (CadF) resulted in increased sIgA reactivity against CadF-coated plates, whereas birds receiving r*L. lactis* (Hcp) and r*L. lactis* (JlpA) showed enhanced antibody responses toward Hcp and JlpA antigens in intestinal lavage, respectively. A similar result was observed with CadF-coated plates for fecal samples, indicating that oral administration of r*L. lactis* expressing *C. jejuni* antigens effectively stimulated antigen-reactive mucosal IgA responses compared with the vector control (Fig. S5, Supplementary information).

### sIgA-mediated in vitro neutralization of *C. jejuni* adhesion

A neutralization assay was performed to assess the in vitro inhibitory effect of intestinal secretory IgA (sIgA) on *C. jejuni* adherence and invasion in CEICs. Direct comparison of total CFU of *C. jejuni* present in infected chicken embryo intestinal cells (CEICs) suggests that, compared to the individual antigen and control (WT *L. lactis*), the sIgA present in the birds immunized with all three antigens significantly inhibits the host cell adhesion and invasion of *C. jejuni* (immunized vs. control) (*p* = 0.0079) (Fig. 2C).

### In vitro stimulation of splenic lymphocytes triggers cell proliferation and NO production

In response to in vitro stimulation with WCL, splenocytes from birds immunized with multicomponent proteins (CadF, JlpA, and Hcp) exhibited significantly higher nitric oxide production in their culture supernatants compared to those immunized with individual proteins or empty NZ9000 cells (immunized vs. control) (*p* = 0.001). In contrast, birds treated with empty NZ9000 cells showed only a basal level of NO production (Fig. 2D).

**Fig. 3:**
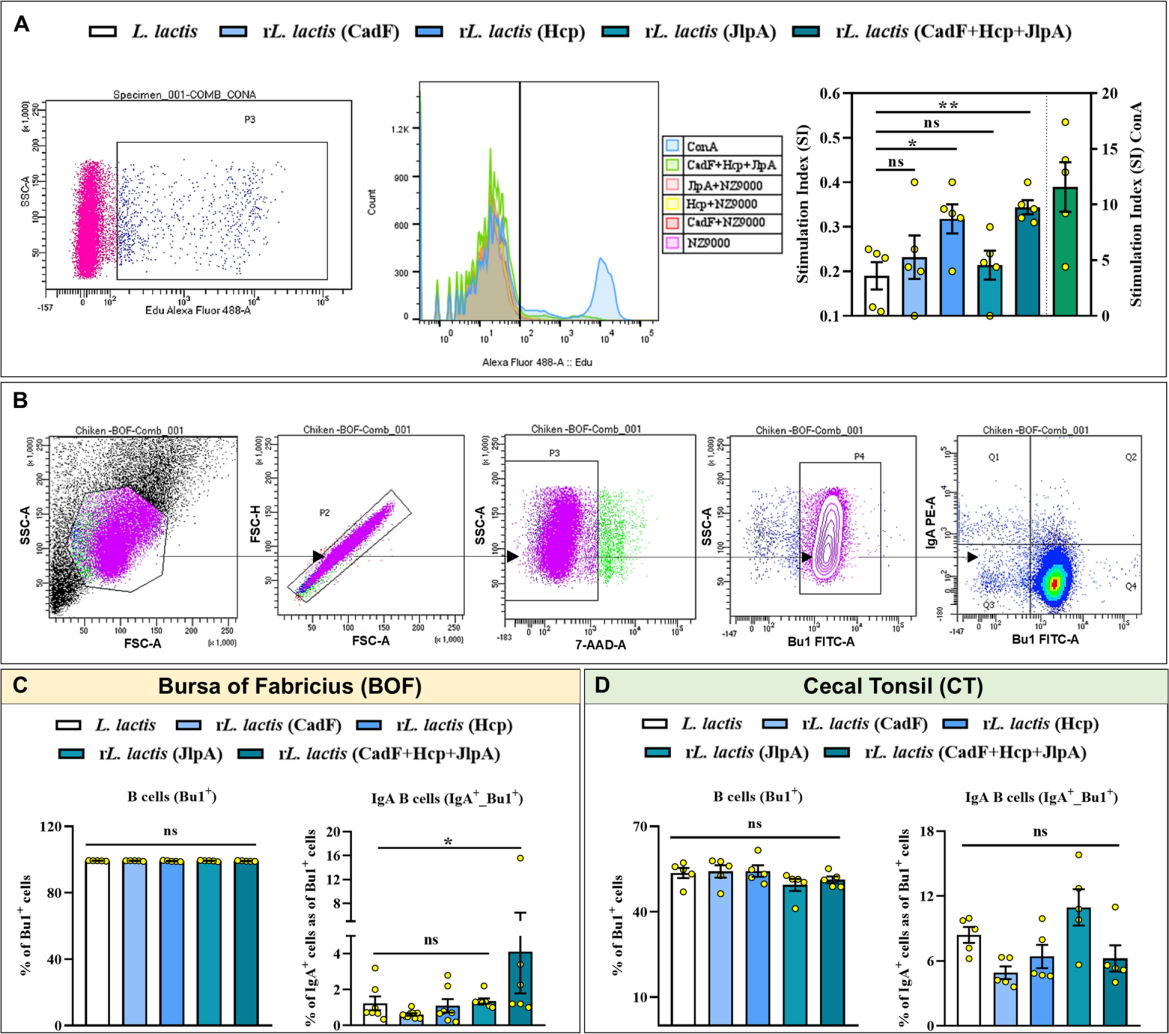
In vitro splenocyte proliferation by in situ EDU assay and flow cytometry analysis of B cell population in BOF and cecal tonsils. **A** In vitro splenocyte (lymphocyte) proliferation by *C. jejuni* whole cell lysate (WCL) (1 μg/mL of WCL) was measured by EDU assay performed at day 21 post first administration, showing a significant difference among r*L. lactis* (CadF + Hcp + JlpA) compared to control groups (WT *L. lactis* only)*.* Data represent mean stimulation index ± SE (*n* = 5). Asterisks indicate a statistically significant difference (**p < 0.01) from control (WT *L. lactis*). **B **Panel showing gating strategy for IgA-positive B cells (Bu1^+^ IgA^+^) present in the Bursa of Fabricius (BOF) and cecal tonsil (CT) of experimental birds. **C**, **D **Flow cytometric analysis of IgA-positive B cells (Bu1^+^ IgA^+^) suggests a modest increase in the birds administered with r*L. lactis* (CadF, JlpA, and Hcp) compared to other experimental groups; however, no such changes could be observed in B cells present in CT

The proliferation of specific splenocytes was significantly higher in birds immunized with the combination of all three antigens than in those receiving individual antigens or the control group (NZ9000) (immunized vs. control) (*p* = 0.004), according to the splenocyte stimulation index (Fig. 3A).

### Change in the B and T cell phenotypes

Mononuclear cells were isolated from BOF, stained, and analyzed by flow cytometry using the gating strategy shown in Fig. 3B. Compared with the control groups, a moderate increase in total IgA-producing B cells (Bu1^+ ^IgA^+^) was observed in the BOF of chickens that received r*L. lactis* expressing the combination of CadF, JlpA, and Hcp (*p* = 0.046) (Fig. 3C). However, no significant differences were observed in IgA-expressing B-cells in the cecal tonsils of the birds (*p* = 0.15) (Fig. 3D). In contrast, we did not observe any changes in T-cell populations (CD3^+^, CD4^+^, and TCRγδ^+^) across groups (Fig. S6, Supplementary information).

### Induction of strong cellular responses in immunized birds

**Fig. 4:**
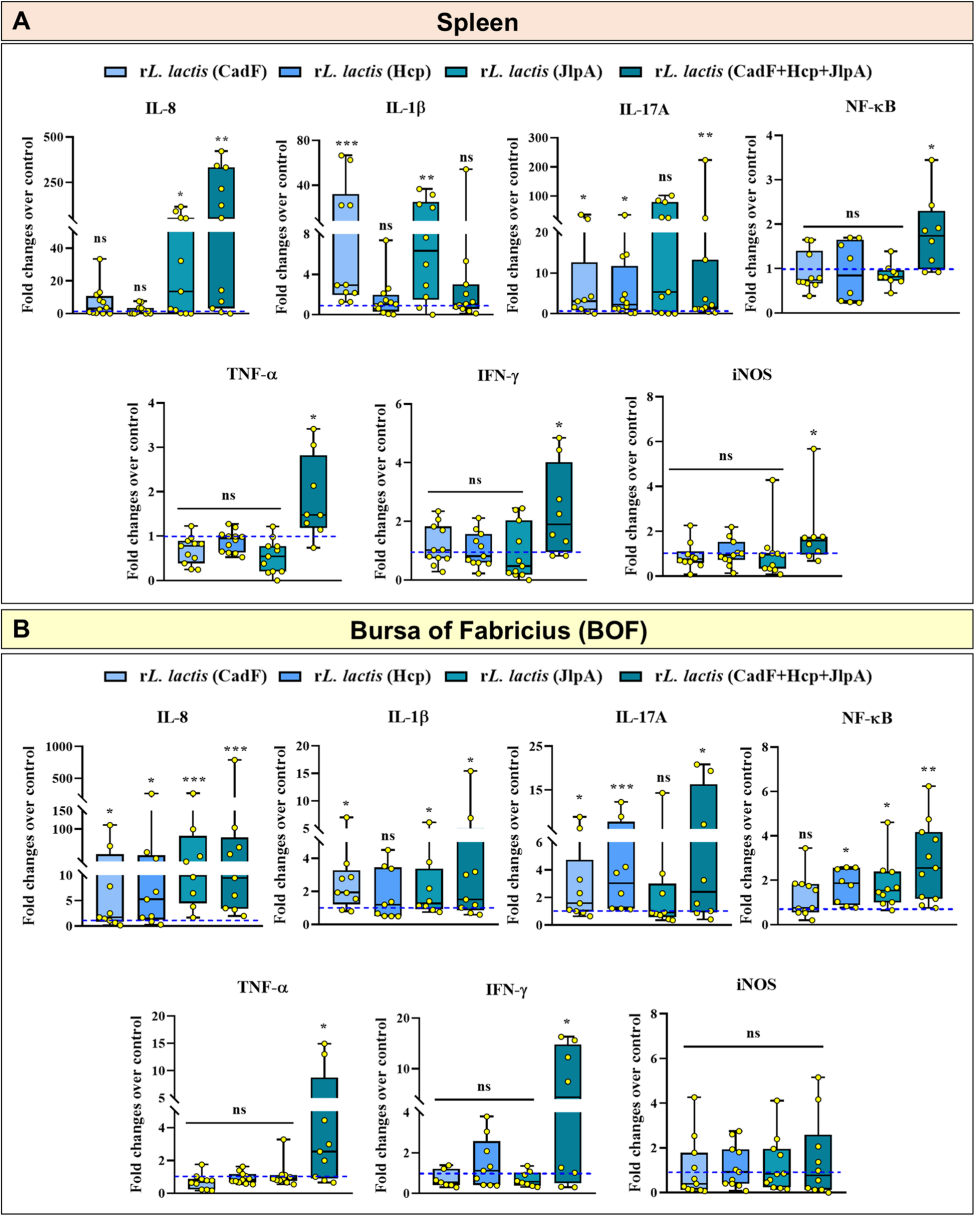
Cytokine gene expression profile in splenic and bursal tissues. **A** Spleen tissues collected on day 28 were subjected to mRNA extraction to analyze the gene expression profile using qRT-PCR. Data show significant upregulation of IL-8, IL-17A, TNF-α, NF-κB, IFN-γ, and iNOS genes in birds that received the combination of all three proteins r*L. lactis* (CadF + Hcp + JlpA). Data represent the mean fold changes of mRNA expression over control (*L. lactis*) (dotted blue line) ± SE of 12 birds/group from two independent experiments (*n* = 12). Outliers were detected and excluded using the ROUT method (*Q* = 1%). **B **Bursa tissue was also collected on day 28, and mRNAs extracted from bursal tissue showed significant upregulation of IL-8, IL-1β, IL-17A, TNF-α, IFN-γ, and NF-κB genes in r*L. lactis* (Cad + Hcp + JlpA) group. Fold changes were calculated with respect to the control group (received WT *L. lactis* only). Data represent the mean fold changes of mRNA expression over control (*L. lactis*) (dotted blue line) ± SE of 12 birds/group from two independent experiments (*n* = 12). Outliers were detected and excluded using the ROUT method (*Q* = 1%)

A critical analysis suggests upregulation of major pro-inflammatory cytokines and transcription factors (IL-8, IL-1β, IL-17A, TNF-α, IFN-γ, and NF-κB) in Splenic and BOF tissues of birds mucosally administered with multicomponent vaccines (CadF, JlpA, and Hcp) compared to birds that received empty *L. lactis* NZ9000 cells (Fig. 4A, B).

### Significant decrease in the cecal load of *C. jejuni* in birds

**Fig. 5:**
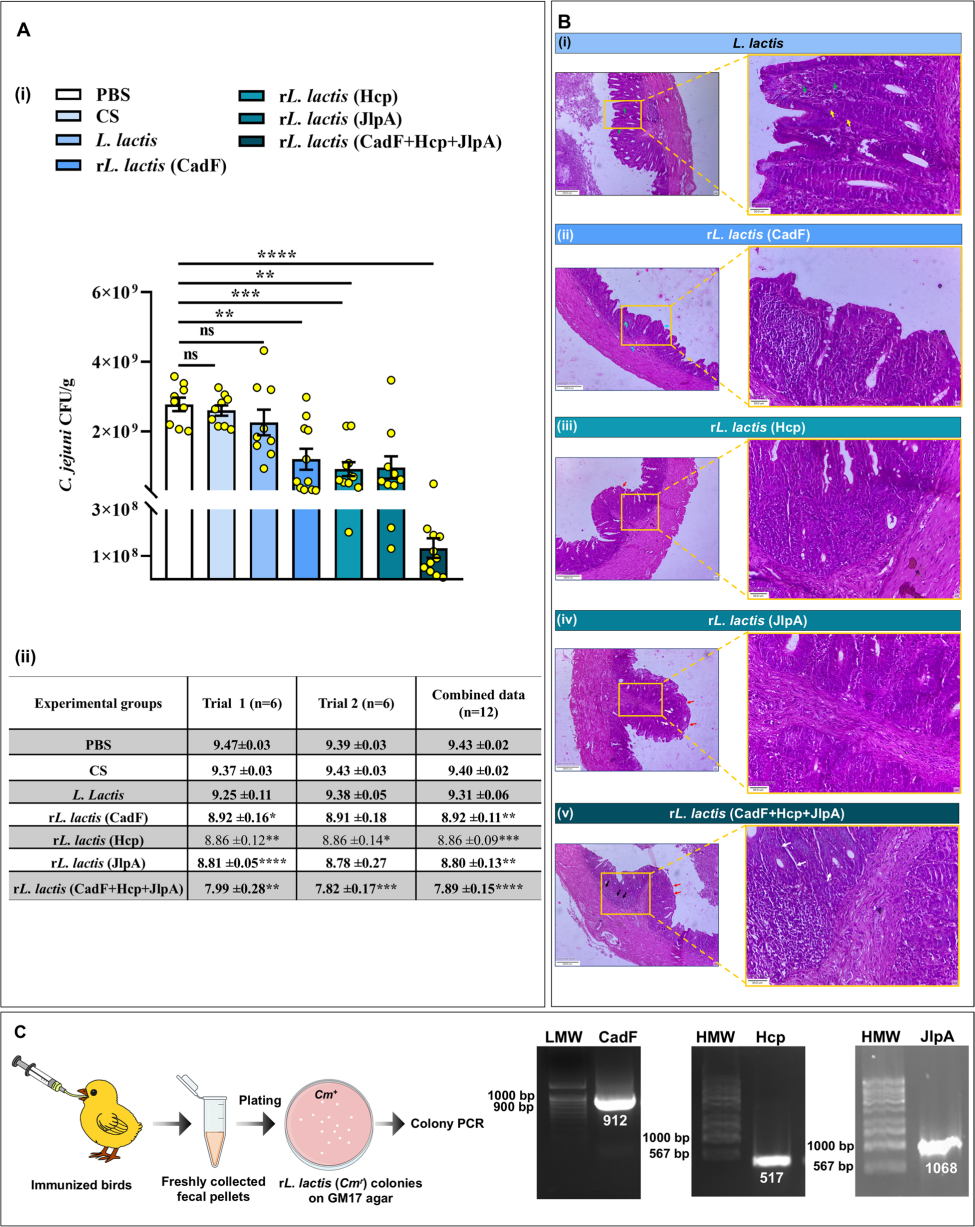
Reduction in cecal load of *C. jejuni *and histopathological analysis of cecal tissue. **A** Birds administered with different combinations of r*L. lactis* were challenged with 1 × 10^8^ CFU *C. jejuni* TGH 9011. On day 7, post-challenge cecal contents were collected and processed to determine the cecal load of *C. jejuni*. Comparative analysis of bacterial load among different experimental groups shows a significant reduction in the birds that received r*L. lactis* expressing CadF, Hcp, and JlpA (alone or in combination) compared to the control group (PBS only). Data represent the (i) mean CFU/gm ± SE and (ii) mean log_10_ CFU/gm ± SE of two independent experiments (*n* = 12) performed under similar conditions. Asterisks indicate a statistically significant difference compared to the PBS group (****p < 0.0001). **B **Histopathological analysis of formalin-fixed paraffin-embedded sections of the cecal tissue collected from birds at day 7 post-infection with *C. jejuni* TGH 9011. The different coloured arrows indicate outcomes in histopathology. Yellow arrow- necrosis, green arrow- infiltration, cyan arrow- reduced edema, light green arrow- reduced infiltration, red arrow- intact epithelia, white arrow- well-structured crypt, black arrow- lymphoid accumulation. **C **Plasmid stability during gut transit. Fresh fecal pellets were collected from immunized birds and plated onto GM17 agar supplemented with chloramphenicol (20 μg/mL). In all cases, bacterial colonies were observed, and upon colony PCR, the target genes were successfully amplified

To assess the effect of mucosal administration of r*L. lactis* expressing CadF, Hcp, and JlpA, the cecal load of each bird was measured on day 7 post-challenge with *C. jejuni*. The results indicate a significant reduction in the bacterial load in the cecum (~ 1.54 log_10_) in immunized birds receiving the combination of all three proteins compared to those receiving individual proteins or the control group (immunized vs. control) (*p* < 0.0001) (Fig. 5A(i–ii)).

### Minimal gut lesions observed in vaccinated birds after *C. jejuni* challenge

Finally, tissue sections from each group were subjected to histopathological analysis to assess the effect of vaccination on preserving gut health in cecal tissue against *C. jejuni* challenge infection. Birds that received the empty vector (*L*. *lactis* only) exhibited significant infiltration of pro-inflammatory cells, particularly heterophils and lymphocytes, within the cecal mucosa and submucosa. These birds also showed clear evidence of edema and blood vessel congestion. The typical architecture of the cecum was severely disrupted, with signs of mucosal erosion, ulceration, and crypt distortion, which indicated compromised epithelial integrity (Fig. 5B(i)).

In contrast, birds immunized with individual proteins or in combination demonstrated markedly improved gut health at post-challenge time points (Fig. 5B (ii, iii, iv)). However, immunization with CadF alone resulted in only mild mucosal erosion (Fig. 5B (ii)). Notably, the combination vaccination with CadF, JlpA, and Hcp provided complete protection of the cecal tissue. Birds in this group displayed well-preserved cecal mucosal architecture, intact epithelial lining, organized crypts, and visible lymphoid aggregates. There was no evidence of mucosal erosion, ulceration, necrosis, or hemorrhage, suggesting that the combined vaccine offers the highest level of protection against *C. jejuni* challenge (Fig. 5B (v)) (Fig. S7, Supplementary information).

### Analysis of gut (cecal) microbial composition in immunized birds

#### Relative abundance of top 10 phyla

Full-length 16S rRNA gene sequencing using the MinION nanopore long-read sequencer revealed significant variation in the cecal microbiota across different experimental groups. The taxonomic profiling at the phylum level among the top 10 most abundant taxa reveals a predominance of three phyla: Bacillota (formerly *Firmicutes*), Pseudomonota, and Bacteroidota. Bacillota remains the most dominant phylum, followed by Pseudomonata and Bacteroidota. Analysis of the top 10 most abundant phyla revealed distinct patterns across experimental groups. Notably, Bacillota, a phylum commonly associated with gut health and metabolic homeostasis, showed consistently high abundance among all groups. The baseline group (day 7 birds) exhibited the highest abundance of Bacillota (~ 98.27%), followed by the UU group (~ 97.94%). Among the vaccinated groups, birds administered with r*L. lactis* (Hcp) showed the highest abundance of Bacillota (~ 94.06%). Interestingly, Bacteroidota, another important phylum involved in carbohydrate fermentation and gut barrier integrity, was most abundant (~ 2.33%**)** in birds that received a combination of all three recombinant *L. lactis* strains (expressing CadF, Hcp, and JlpA). This enrichment may indicate some synergistic benefits of the current vaccine composition on gut microbial balance.

**Fig. 6:**
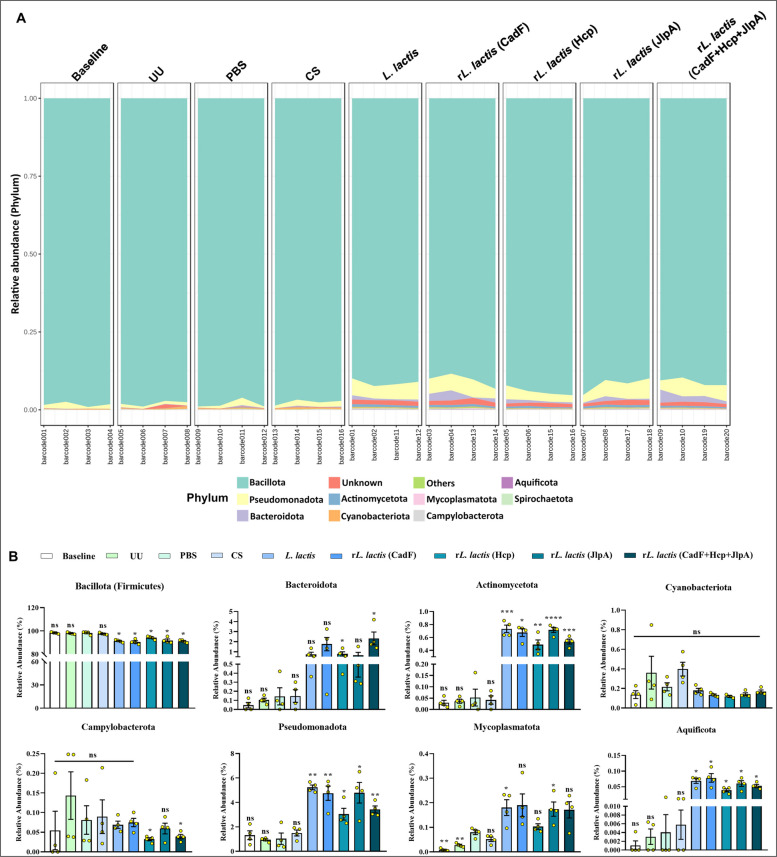
Relative abundance of cecal microbiota at the phylum level. **A **The relative abundances of cecal microbiota at the phylum level in birds with different r*L. lactis* treatments based on full-length 16S rRNA gene sequencing are shown as stacked area plots, *n* = 4 per group. **B **The relative abundances (%) of the top 10 phyla in the cecal microbiota of different birds showed that Bacillota was the top phylum and showed steadily high abundance among all groups. Bacteroidota was abundant in birds receiving a combination of all three recombinant *L. lactis* strains (expressing CadF, Hcp, and JlpA). r*L. lactis* combination led to a reduction in the relative abundance of Campylobacterota. No significant differences were observed across groups in the abundance of other major phyla, including Cyanobacteriota and Mycoplasmatota

In contrast, administration of the recombinant *L. lactis* cocktail (CadF + Hcp + JlpA) led to a ~ 2-fold reduction in the relative abundance of Campylobacterota compared to the PBS control, a phylum known for its pathogenic and opportunistic bacteria. Moreover, significant differences were observed among the phyla Actinomycetota, Pseudomonadota, and Aquificota. However, no significant differences were observed across groups in the abundance of other major phyla, including Cyanobacteriota and Mycoplasmatota, indicating that the probiotic intervention did not broadly disrupt the existing gut microbial community structure (Fig. 6A, B) (Table S5, Supplementary information).

#### Relative abundance of top 20 genera

**Fig. 7:**
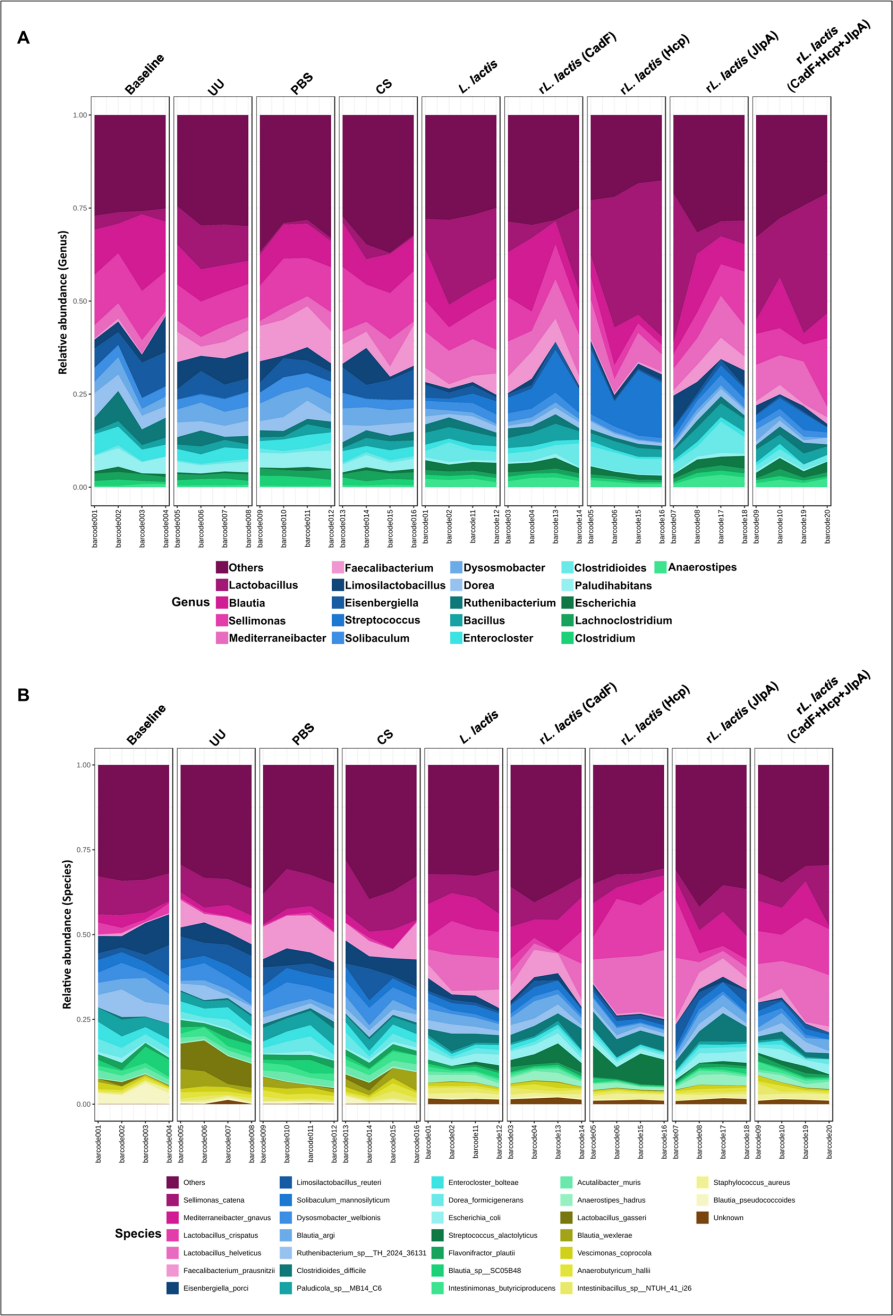
Relative abundance at the genus and species level of cecal microbiota. **A **The relative abundances of the top 20 genera in the cecal microbiota of the birds based on full-length 16S rRNA gene sequencing are shown as stacked area plots. Birds immunized with r*L. lactis* expressing Hcp, and those receiving the combination of all three recombinant strains (CadF + Hcp + JlpA) exhibited the highest relative abundance of *Lactobacillus* compared to the control group. **B **The relative abundance of the top 30 species in the cecal microbiota of the birds is shown in stacked area plots. *Lactobacillus helveticus* and *Lactobacillus crispatus* appeared to be the most abundant species in the *Lactobacillus* genus in the Hcp and combined (CadF + Hcp + JlpA) group

Further taxonomic analysis at the genus level revealed that *Lactobacillus* was the most dominant genus within the phylum Bacillota, with notable variation across experimental groups. In particular, birds immunized with r*L. lactis* expressing Hcp, as well as those receiving the combination of all three recombinant strains (CadF + Hcp + JlpA), exhibited the highest relative abundance of *Lactobacillus* (~ 29%)**,** in contrast to a lower abundance in the *L. lactis* control group (~ 17%). Other genera among the top 20 most abundant included *Blautia, Sellimonas, Mediterraneibacter*, *Faecalibacterium*, *Limosilactobacillus*, *Eisenbergiella*, *Streptococcus*, and *Solibaculum*. However, these genera did not exhibit substantial differences in abundance across experimental groups (Fig. 7A).

#### Relative abundance of top 30 species

At the species level, analysis of the top 30 most abundant taxa further clarified the specific microbial shifts associated with recombinant *L. lactis* immunization. *Sellimonas catena* and *Mediterraneibacter gnavus* had the highest relative abundance among the species. Within the dominant genus *Lactobacillus,* *Lactobacillus helveticus* and *Lactobacillus crispatus* emerged as the most abundant species**,** particularly in the Hcp-immunized and combined antigen (CadF + Hcp + JlpA) groups (Fig. 7B).

### Alpha and beta diversity metrics of the cecal microbiota

#### Alpha diversity

**Fig. 8:**
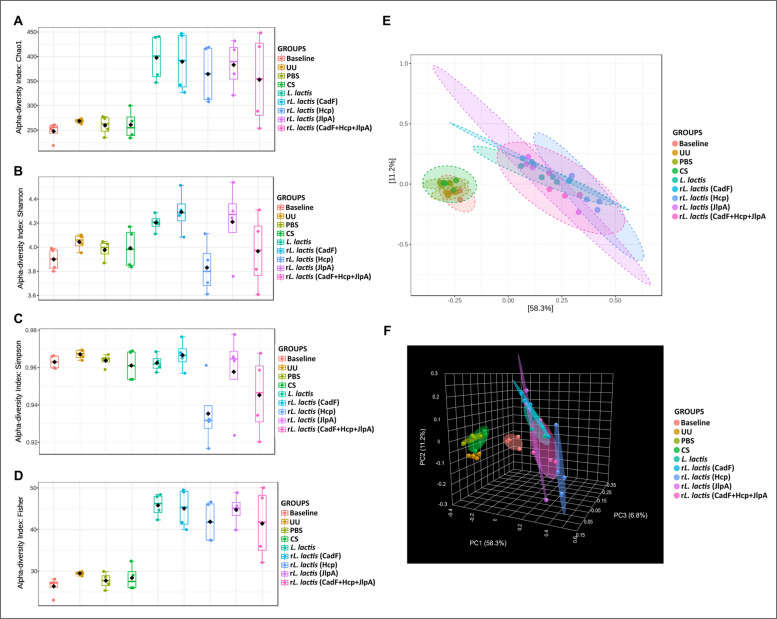
Alpha and beta diversity matrices among groups. **A**–**D **Alpha diversity matrices of the cecal microbiota of birds were evaluated using Chao1, Shannon, Simpson, and Fisher diversity indexes. The Chao1 index was used to assess the species richness. The microbial communities’ evenness and richness were both reflected in the Shannon index. The Simpson index highlighted dominance, and with less impact from evenness, Fisher’s alpha displayed the species richness. **E**, **F **Beta diversity was assessed with the Bray-Curtis dissimilarity index and statistically tested by the PERMANOVA test (*p* < 0.05) and represented by the principal coordinates analysis (PCoA) plot and 3D plot

Alpha diversity matrices (Chao1, Shannon, Simpson, and Fisher indexes): Chao1 estimates taxa richness by accounting for undetected features because of low abundance. Shannon and Simpson consider species richness and evenness, with varying weight given to evenness, and Fisher models the community abundance structure as a logarithmic series distribution. Alpha diversity, as revealed by Chao1 and Fisher indices, was significantly higher in all vaccinated groups compared to the unvaccinated control and baseline, suggesting greater species richness. Alpha diversity, measured by the Shannon index, was higher in the *L. lactis* and r*L. lactis* (CadF) and r*L. lactis* (JlpA) groups compared to baseline, while r*L. lactis* (Hcp) showed a lower mean Shannon index, despite elevated Chao1, suggesting higher species richness at the expense of reduced evenness. Consistent with the *Lactobacillus* enrichment observed at the genus level, Simpson index values were slightly lower in the r*L. lactis* (Hcp) and r*L. lactis* (CadF + Hcp + JlpA) groups compared to baseline. An increase in the relative abundance of a single species might increase species richness but decrease the Simpson index (Fig. 8A–D) (Table S6, Supplementary information).

#### Beta diversity and community structure shifts

To assess overall differences in microbial community composition among the treatment groups, Principal Coordinates Analysis (PCoA) was performed based on Bray–Curtis dissimilarity, with statistical significance confirmed using PERMANOVA with an FDR correction (Benjamini-Hochberg). The resulting ordination plot revealed distinct clustering patterns among the experimental groups. Birds in the control groups clustered together, suggesting similar community structure. In contrast, birds receiving *L. lactis* or r*L. lactis* recorded significant shifts (*p* < 0.05) away from the controls (Fig. 8E, F) (Table S7, Supplementary information).

### Differential abundance analysis of microbiota in birds

To identify which microbial groups were uniquely enriched in the experimental groups, a linear discriminant analysis effect size (LEfSe) analysis was conducted at the genus and species levels, using linear discriminant analysis (LDA) scores to identify potential microbial biomarkers. The LEfSe analysis showed distinct microbial shifts across the different *L. lactis* treatment groups. At the genus level, *Lactobacillus* (LDA > 5.0) was the most robustly discriminating taxon, with the highest abundance in all r*L. lactis* groups, especially the r*L. lactis* (Hcp) and (CadF + Hcp + JlpA) group. Genera *Streptococcus*, *Sellimonas*, *Faecalibacterium*, *Blautia*, *Eisenbergiella,* and *Mediterraneibacter* were all differentially enriched in the LAB-treated groups compared to the baseline and PBS controls.

**Fig. 9:**
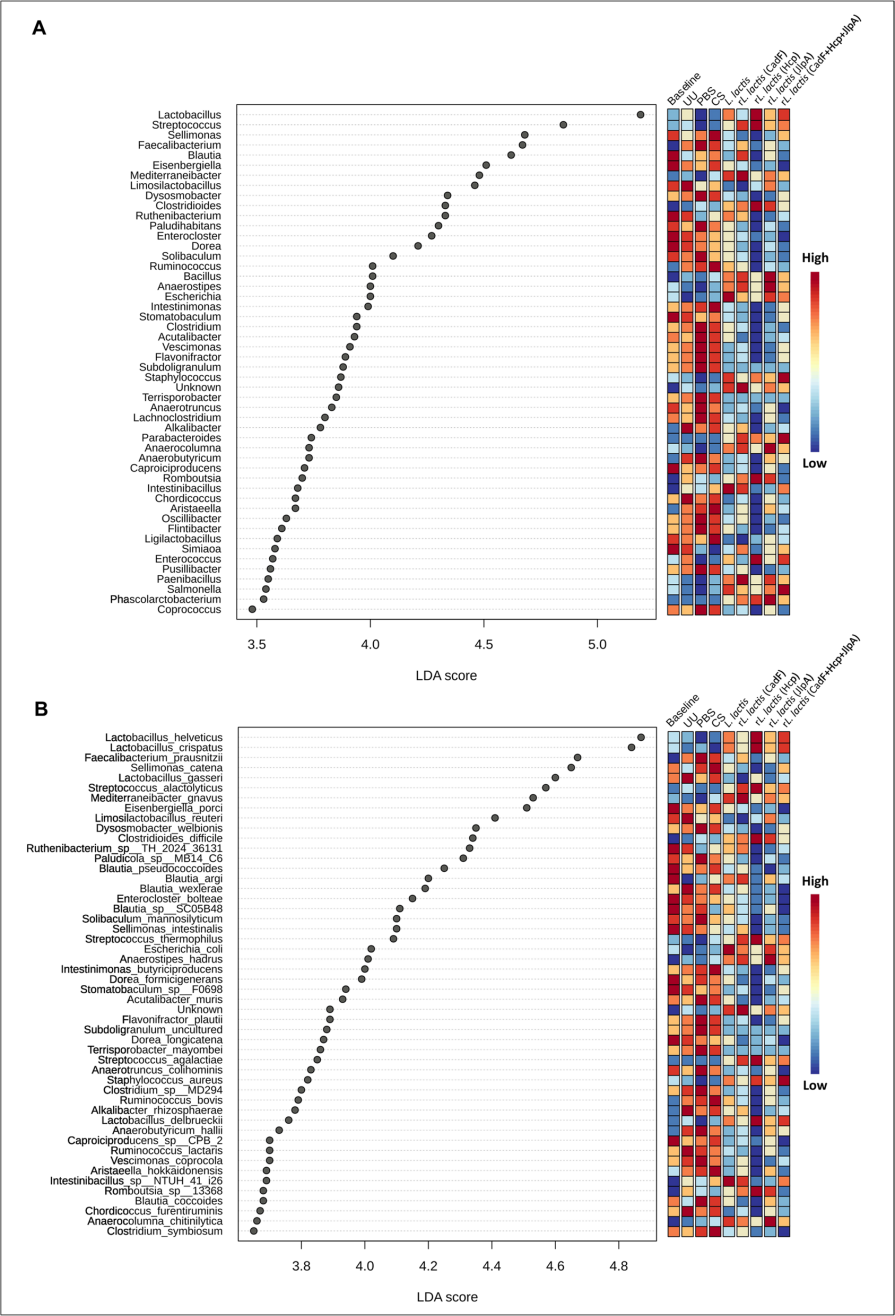
Differential abundance analysis and microbial cluster patterns among groups. **A**, **B** Differential abundance analysis comparing bacterial taxa in the cecum of experimental birds with different treatments. The dot plot shows the results of the LEfSe analysis, highlighting the significantly different taxa at the genus and species levels (LDA score > 2, *p* < 0.05) in the cecal microbiota of birds

LEfSe analysis and associated genus-level heatmaps revealed distinct microbial signatures across treatment groups. For example, birds administered with r*L. lactis* groups were characterized by an enriched level of lactic acid bacteria, including *Lactobacillus* spp., compared to control groups. However, we observed an increase in *Escherichia* and *Clostridioides* in r*L. lactis*-Hcp and -JlpA-expressing administered birds (Fig. 9A). The exact reason for the observed enrichment of *Escherichia* and *Clostridioides* remains unclear.

At the species level, distinct microbial signatures were observed across the treatment groups, mirroring the genus-level patterns. *Faecalibacterium prausnitzii*, *Sellimonas catena*, *Lactobacillus gasseri*, and *Streptococcus alactolyticus* were all differentially enriched in the LAB-treated groups. Additionally, *Lactobacillus helveticus* and *Lactobacillus crispatus* (LDA > 4.5) were recorded as the dominant discriminating taxa in the LAB-treated groups (Fig. 9B).

### Microbial composition and cluster patterns across treatment groups

The heatmap showed the relative abundance of microbial genera between treatment groups and corroborated the PCoA report. Similar to the PCoA plot, two major clusters were observed: the baseline/control cluster group and the *L. lactis* and r*L. lactis* groups, which showed higher abundances of *Lactobacillus*, *Limosilactobacillus*, *Sellimonas*, and *Faecalibacterium*. Within the LAB-treated group cluster, two subclusters are observed, including the r*L. lactis* (CadF + Hcp + JlpA) and r*L. lactis* (JlpA) groups and the r*L. lactis* (CadF) and r*L. lactis* (Hcp) group.

The *L. lactis* group showed a distinctive microbial signature, with relatively intermediate abundance patterns across most taxa. The combined vaccinated group of birds exhibited the most distinctive microbial profile, characterized by a heavy enrichment or depletion of several genera relative to the *L. lactis* group. Significant divergence from the *L. lactis* group was also observed in the r*L. lactis* (Hcp) and r*L. lactis* (JlpA) groups, which formed separate clusters and displayed differential abundance patterns with selectively enriched red and depleted blue areas, indicative of selective enrichment or suppression of important bacterial taxa. Group-specific changes in abundance were observed in species such as *Lactobacillus*, *Clostridium*, *Ruminococcus*, and *Bacteroides*, with specific genera steadily enriched or depleted by the present vaccine composition, highlighting the impact of antigen-specific immune modulation on gut microbial dynamics (Fig. S8, Supplementary information).

## Discussion

Together with the emergence of new drug-resistant strains of *C. jejuni* and their intrinsic ability to colonize the chicken gut pose substantial risks to food safety and public health [44–46]. On the other hand, vaccination against *C. jejuni* remains challenging due to several key factors, including its high genetic diversity, frequent phase variation, extensive serotype diversity, and immune evasion [47–49]. Given that *C. jejuni* colonization is a multifactorial process, one approach to address these challenges is to design a multicomponent vaccine using immunogenic subunits with high sequence conservation across diverse *C. jejuni* strains. To this end, several surface-exposed and secretory proteins of *C. jejuni* have been identified as potential vaccine targets due to their sequence conservation and roles in bacterial adherence, invasion, and virulence. Among these proteins, Hcp, JlpA, CadF, and FlpA, along with other major outer membrane proteins (MOMPs) like PorA, CmeC, and PEB1, have emerged as promising vaccine candidates for controlling *C. jejuni* infections [17, 20, 32, 49–51]. Specifically, the *C. jejuni* CadF and JlpA proteins are highly conserved key SECPs that mediate host cell adherence by binding to fibronectin (FN) and the tissue chaperone Hsp90α, respectively. These interactions represent crucial early steps in bacterial colonization to trigger pro-inflammatory host responses [52–54].

Similarly, as a critical effector protein of the functional Type VI secretion system (T6SS), *C. jejuni* Hcp can contribute to bacterial competition and environmental adaptation. Recently, we demonstrated that Hcp enhances competitive fitness in a dynamic gut environment, and mucosal delivery of recombinant Hcp can drive robust immune responses within the intestinal mucosa in avian and murine models [15, 39]. Thus, with its dual roles in pathogenesis and immunogenicity, and given the high sequence conservation among *C. jejuni* strains, Hcp can serve as a key candidate for a combinatorial vaccine. Moreover, our in silico studies identified multiple conserved putative B-cell epitopes within the sequences of CadF, Hcp, and JlpA, strengthening their potential as promising multicomponent vaccine targets to limit the cecal load of *C. jejuni* in chickens [15, 16]. The utility of SECPs and effector proteins of *C. jejuni* as vaccine targets has been demonstrated using OMV-based vaccine platforms; however, their application is limited by the presence of lipooligosaccharide (LOS) and the associated risk of endotoxin-mediated reactogenicity [34, 55].

To this end, the present study aims to evaluate the prospective benefits of a safe mucosally deliverable multicomponent vaccine strategy incorporating three immunogenic proteins (CadF, Hcp, and JlpA), each with distinct functions, to determine whether this combination offers superior protection compared to a monovalent approach.

For this purpose, we used bioengineered recombinant *L. lactis* surface-expressing CadF, Hcp, and JlpA proteins of *C. jejuni*. To enhance mucosal adherence, intragastric stability, and prolong gut transit time, we further coated the recombinant bacteria with chitosan. As a natural biopolymer, chitosan is a linear cationic polysaccharide consisting of N-acetyl-d-glucosamine and d-glucosamine coupled by β (1 → 4) glycosidic linkages. This conformation enables chitosan to bind sialic acid and other negatively charged biomolecules in mucus and epithelial cell surfaces through electrostatic interactions. Additionally, incorporating sodium tripolyphosphate (TPP), a non-toxic polyanionic salt, facilitates cross-linking of chitosan, thereby enhancing the gelation and reticulation capacity of the resulting biopolymer [56–59]. Previously, we showed that CS-TPP coating of the LAB vector does not adversely affect bacterial growth; rather, it acts as a protective barrier against a range of physicochemical conditions mimicking the gut [29]. We confirmed that the CS coating of *L. lactis* cells does not affect the accessibility of the CadF, Hcp, and JlpA proteins expressed on the bacterial surface. Furthermore, in vitro cell adhesion studies supported our hypothesis that chitosan coating enhances bacterial cell association due to its strong mucoadhesive properties [60–62]. This observation aligns with our previous findings, which demonstrated that chitosan coating can extend the gut transit time of LAB vectors in both murine and avian models [29]. Moreover, recovery of recombinant LAB vector from fecal samples of orally administered birds confirmed plasmid stability during gut transit (Fig. 5C). These observations have remained a strong foundation for us to explore the in vivo application of non-commensal LAB strains, such as *L. lactis*, as live vector vaccine delivery platforms against *C. jejuni.*

We showed that oral administration of chitosan (CS)-coated recombinant *L. lactis* expressing the three antigenic proteins (CadF, Hcp, and JlpA) resulted in a significant increase in antigen-specific local antibody levels, specifically secretory IgA (sIgA) against *C. jejuni* in both intestinal lavages and freshly collected faecal pellets. Compared to immunization with individual antigens, enhanced mucosal antibody responses by the combined delivery of all three subunits suggest the more pronounced effect of the “combinatorial approach” at the mucosal surface. In contrast, oral administration of r*L. lactis* did not influence systemic antibody (IgY) levels, likely because mucosally activated IgY-producing B cells do not readily traffic into the systemic circulation [15, 16]. Nevertheless, both unimmunized control groups (PBS or Chitosan) of birds failed to elicit local antibody responses; therefore, we did not include them in further immunological assessment, except for cecal load determination; instead, comparisons were made with the CS-coated WT *L. lactis*. Importantly, we also demonstrated that a cocktail of antibodies harvested from intestinal lavages neutralized *C. jejuni* adherence and invasion of primary CEICs. These findings further confirm that local antibodies raised by mucosal delivery of r*L. lactis* expressing CadF, Hcp, and JlpA can specifically recognize and bind their corresponding surface antigens on *C. jejuni*, thereby interfering with bacterial colonization and invasion.

Since vaccine efficacy depends on the induction of both humoral and cellular immune responses, we evaluated cellular immune responses in peripheral lymphoid organs to assess the potential of the current vaccine formulation to promote long-term immunity, memory formation towards orchestration of antibody production. To this end, our immunophenotyping data indicate a synergistic effect in IgA^+^ B cell populations in the Bursa of Fabricius (BOF) of birds immunized with the combination of all three antigens, compared with those immunized with a single antigen. However, no noticeable changes were observed in the CD3⁺, CD4⁺, and TCRγδ⁺ T cell populations in the cecal tonsils of immunized birds (Fig. S6, Supplementary information). This may be due to the nature of the current mucosal vaccine modality, which uses recombinant LAB vectors and appears to be less effective at inducing T cell differentiation, instead preferentially activating B cells to provide immediate mucosal protection. While the humoral and cellular arms of the immune system are inherently interconnected, our findings further highlight a synergistic enhancement of cellular immunity in birds vaccinated with the combined antigen formulation (CadF, Hcp, and JlpA). Specifically, birds that received combined vaccination exhibited a marked splenocyte proliferative response and increased nitric oxide (NO) production upon exposure to *C. jejuni* whole-cell lysate, indicating activation of immune effector functions. Notably, increased cell proliferation and NO production correlated with concurrent upregulation of key pro-inflammatory and immune-regulatory genes, including IL-8, IL-1β, IL-17A, TNF-α, and NF-κB, in birds receiving r*L. lactis*. The involvement of NF-κB, a central transcription factor in inflammation and host defense, further supports the immunostimulatory potential of this vaccine strategy [63, 64]. However, protein-level validation of these immune-regulatory genes would strengthen the findings beyond transcriptional analysis. Together, we conclude that recombinant *L. lactis*-based mucosal immunization, particularly with multiple antigens, can elicit both innate and Th1-type systemic immunity.

We next asked whether a Th1-biased response and potent neutralizing antibodies can contribute to restricting bacterial colonization in immunized birds. We therefore performed an in vivo challenge experiment with highly pathogenic *C. jejuni* (TGH 9011) [65, 66]. The results from our challenge experiment demonstrating > 1.5 log_10_ reduction in cecal load of *C. jejuni* strongly support the efficacy of the multicomponent vaccine composition in limiting bacterial colonization in immunized birds (Fig. 5A (i–ii)). Moreover, histopathological analysis of the cecal tissue from vaccinated birds challenged with *C. jejuni* showed well-preserved intestinal architecture, with intact villi, minimal infiltration of inflammatory cells, and reduced epithelial damage, compared with unvaccinated controls, which showed marked villus blunting, mucosal erosion, and lymphocytic infiltration (Fig. 5B). Together, these findings suggest that the multicomponent vaccine reduced pathogen burden, mitigated gut inflammation, and maintained mucosal integrity. In line with our observation, several *L. lactis* strains, including the one used in this study (NZ9000), have demonstrated mucus-binding, antimicrobial, and immunomodulatory properties, including the enhancement of barrier integrity [67, 68]. These properties are associated with beneficial gut-targeted activities that may enhance microbial fitness and persistence within the host gastrointestinal tract [69].

Vaccination can significantly impact poultry health both positively and, in some cases, negatively, depending on the route of administration, vaccine composition, dosage, and vaccination regimen. In commercial poultry production systems, the vaccination approach must aim to enhance immunoprotection and improve productivity, particularly growth rate, feed conversion efficiency, and egg production [70, 71]. Given these considerations, we selected probiotic bacteria as a delivery platform for the present vaccine formulation, anticipating that this approach would offer dual benefits: enhanced immune protection and improved gut health.

The analysis of full-length 16S rRNA gene sequencing of the cecal microbial community highlighted the predominance of Bacillota (formerly *Firmicutes*) and Bacteroidota, suggesting that immunization may have modulated the gut microbiota in a favorable direction, contributing to improved gut health, as corroborated by histopathological findings. In particular, the elevated abundance of Bacteroidota in the immunized group points to a possible selective expansion of beneficial microbial populations. Additionally, a significant reduction in the relative abundance of Campylobacterota in either the r*L. lactis* (Hcp) or the combined treatment group indicates depletion of the harmful microbiota. These findings support the notion that, in addition to enhanced immunoprotection, administering r*L. lactis* strains may exert additional protective effects by promoting the growth of selected beneficial microbes while suppressing potentially harmful populations (Fig. 6A, B) [72–74].

In-depth taxonomic analysis at the genus level further revealed that *Lactobacillus* was the most dominant genus within the Bacillota phylum, with its abundance showing notable variation across experimental groups. The enrichment of *Lactobacillus*, a well-characterized probiotic genus known for enhancing intestinal barrier integrity, competitively excluding pathogens, and modulating host immune responses, reinforces the health-promoting potential of recombinant *L. lactis*, especially when engineered to express immunogenic antigens such as Hcp (Fig. 7A) [75]. The functional roles of *Lactobacillus* in the chicken gut, including fermentation of dietary carbohydrates to produce lactic acid, lowering gut pH, inhibiting pathogens, enhancing mucosal immunity, and modulating host inflammatory responses, particularly under challenge conditions, are well documented [76, 77].

When searching for other top genera, *Blautia*, *Sellimonas, Mediterraneibacter, Faecalibacterium, Limosilactobacillus, Eisenbergiella, Streptococcus,* and *Solibaculum* are of note; however, no noticeable changes could be observed across the vaccinated groups of birds (Fig. 7A). This led us to interpret that the *L. lactis*-mediated influence on gut microbial community structure was selective rather than broad-spectrum, likely driven by specific probiotic-host or probiotic-microbe interaction [78]. While analysing the microbial communities across treatment groups using alpha and beta diversity indices, we observed a significant increase in alpha diversity indices, particularly Chao1 and Fisher indices, in all *L. lactis*-treated groups, suggesting a *Lactobacillus*-enriched cecal community. A richer and more diverse gut microbiota was found to be associated with ecological resilience and competitive-exclusion potential [79]. Specifically, in the context of *C. jejuni* colonization, a *Lactobacillus*-enriched cecal community can confer this competitive exclusion advantage, due to the lactic acid- and bacteriocin-producing abilities of *Lactobacillus* spp. [80]. Furthermore, the observed differences in alpha diversity indices, Chao1/Fisher richness increases, and the variable Shannon/Simpson patterns for r*L. lactis* (Hcp) and r*L. lactis* (CadF + Hcp + JlpA) groups are not suggestive of dysbiosis. Instead, they indicate a *Lactobacillus*-dominant community. The Shannon and Simpson indices are sensitive to the relative evenness of taxa. Because Shannon and Simpson indices reflect evenness, dominance of a single taxon (e.g., *Lactobacillus*, in our case) reduces diversity estimates despite increased species richness.

To further assess beta diversity and shifts in community structure, the resulting ordination plots (Fig. 8E, F) revealed distinct clustering patterns among the experimental groups. Specifically, there was a clear separation between the *L. lactis* -treated (either recombinant or wild type) and the control groups (which received PBS or Chitosan). The separation of all *L. lactis*-administered groups from baseline and control groups, regardless of heterologous antigen expression profile, suggests that the *L. lactis* vector itself, encapsulated in chitosan, is the primary driver of microbiota remodeling. This is consistent with the reported mucoadhesive properties of chitosan, which prolong the retention of encapsulated bacteria in the gut lumen and enhance their interaction with epithelial and immune cells, thereby amplifying the ecological impact of even a non-colonizing vector [81].

To identify specific taxa driving the observed diversities among the experimental groups, we performed microbiota differential abundance testing (LEfSe) analysis, which identified *Lactobacillus* (genus), *L. helveticus*, and *L. crispatus* as key predominant taxa across the vaccinated birds, along with some enrichment of *Faecalibacterium prausnitzii*, a major butyrate-producing commensal linked to gut health. These findings suggest that the r*L. lactis*-based vaccine modulates the gut microbiota by promoting beneficial lactic acid bacteria while selectively restructuring the microbial community. Further, chitosan encapsulation enhances bacterial retention, thereby promoting a more resilient cecal microbiome. However, considering the inherent plasticity of the microbiota, probiotic effects exhibited by non-commensals (*Lactococcus* spp.) or allochthonous (e.g., *Pediococcus acidilactici* and *Saccharomyces cerevisiae* boulardii) are transient and short-term [82, 83]. Nevertheless, we propose that chitosan encapsulation enhances bacterial retention, thereby promoting a more resilient and sustained cecal microbiome composition following vaccination. However, this hypothesis requires dose optimization and validation through long-term studies to confirm the durability of these effects and their functional relevance.

In view of the potential bidirectional interplay between vaccine-induced changes in the microbiota and immune signaling, it would be of interest to investigate whether correlations exist between microbial composition and diversity and the elevated immune status observed in immunized birds [84]. Although we observed some evidence of a correlation between enhanced microbiota diversity, characterized by the predominance of beneficial taxa, and mucosal immunoprotection mediated by elevated sIgA levels in the gut of the birds belonging to the combined vaccine group. Importantly, in terms of bacterial enrichment, we noted that *Lactobacillus* (genus), *L. helveticus*, and *L. crispatus* (species) were the strongest biomarkers at the vaccinated state and also highlighted the enrichment of *Faecalibacterium prausnitzii*. As *F. prausnitzii* is a major butyrate-producing commensal with potent anti-inflammatory properties in both human and animal microbiomes, its abundance in vaccinated birds supports overall improvement of gut health in vaccinated birds compared to unvaccinated controls [85, 86]. This suggests that bidirectional interactions between the gut microbiome and mucosal immunity actively shape microbial community composition, and, in this study, the observed shifts are more likely driven by immune-mediated pathogen clearance than by passive microbial responses [87–89].

Taken together, the comparable alpha and beta diversity, preserved gut histology, and reduced cecal load of *C. jejuni* across the vaccinated groups using r*L. lactis*-based delivery of CadF, Hcp, and JlpA (alone or in combination) suggests that modulation of gut microbial composition is likely functional rather than taxonomic restructuring.

## Conclusions

Our preliminary data, combined with the intrinsic adjuvanticity of the proposed vaccine strategy, underscore its potential to preserve gut homeostasis and safeguard the intestinal mucosa against *C. jejuni* pathogenesis without inducing immune tolerance. Notably, bioengineering probiotic bacteria emerge as a promising live vector-based mucosal vaccine platform against common enteric pathogens, including *C. jejuni*, due to their capacity to stimulate both mucosal and, to some extent, systemic immune responses through the controlled expression of target proteins. Given that the efficacy of mucosal vaccines largely relies on regulating bacterial adhesion and invasion of the intestinal epithelium, optimal protein expression in the complex gut environment is critical to maximizing the adaptability and effectiveness of LAB-based vaccine strategies.

## Supplementary Information

Supplementary Material 1: Table S1. The list of bacterial strains and plasmids. Table S2. Composition of poultry feed used in the present study. Table S3. Detailed list of the antibodies used for flow cytometry. Table S4. Cytokine gene primers used for qRT-PCR and CadF cloning primers. Table S5. Multiple linear regression with covariate adjustment of cecal microbiota at the phylum level. Table S6. Post-hoc pairwise comparison (multi-group) of alpha diversity indexes. Table S7. Pairwise PERMANOVA analysis of beta diversity. Fig. S1. Bioengineering *L. lactis* surface expressing CadF protein of *C. jejuni. *Fig. S2. Standard plot for sodium nitrite (NaNO_2_) using Griess reagent. Fig. S3. In vitro adhesion assay of uncoated and CS-coated r*L. lactis* (CadF, Hcp, JlpA) in CEICs. Fig. S4. SDS-PAGE analysis of recombinant CadF, Hcp and JlpA proteins. Fig. S5. sIgA levels in intestinal lavages and fecal soups against individual proteins by ELISA. Fig. S6. Flow cytometric analysis of T-cell subsets in cecal tonsils of immunized birds. Fig. S7. Additional images of tissue sections showing histopathological changes of cecal tissue collected from birds at day 7 post-infection with *C. jejuni* (TGH 9011). Fig. S8. Clustering heatmap showing relative abundance of microbial genera between treatment groups.

## Data Availability

The authors confirm that the data supporting the findings of this study are available within the article and its supplementary materials. Raw data generated from 16S rRNA gene sequencing have been submitted to the NCBI Sequence Read Archive (SRA) repository under Bioproject accession PRJNA1355441. The raw data from 16S rRNA gene sequencing have also been deposited in Figshare and are accessible via the links: https://figshare.com/s/ae746205f00854da368d and https://figshare.com/s/8878dcbefa6ff4ffa0b1.

## Abbreviations

LMICs: Low- and middle-income countries
SECPs: Surface-expressed colonization proteins
LAB: Lactic acid producing bacteria
CEICs: Chicken embryo intestinal cells
RT: Room temperature
TPP: Sodium tripolyphosphate
RIR: Rhode Island Red
BOF: Bursa of fabricius
WCL: Whole cell lysate
NO: Nitric oxide
EDU: 5-Ethynyl-2′-deoxyuridine
TMB: 3,3′,5,5′-Tetramethylbenzidine
Con A: Concanavalin A,
PFA: Paraformaldehyde
CAT: Cefoperazone, amphotericin B, and teicoplanin
H&E: Hematoxylin and eosin
OTUs: Operational taxonomic units
MOMPs: Major outer membrane proteins
T6SS: Type VI secretion system
