# Supplementary material for: Recombinant LAB vector-based multicomponent vaccine against Campylobacter jejuni potentially promoting a healthier microbial balance in the poultry gut
Source: Microbiome. 2026 May 7;14:174. doi: 10.1186/s40168-026-02421-w (PMC13321747; doi:10.1186/s40168-026-02421-w)
Supplement: Supplementary file 2 — Supplementary Material 1: Table S1. The list of bacterial strains and plasmids. Table S2. Composition of poultry feed used in the present study. Table S3. Detailed list of the antibodies used for flow cytometry. Table S4. Cytokine gene primers used for qRT-PCR and CadF cloning primers. Table S5. Multiple linear regression with covariate adjustment of cecal microbiota at the phylum level. Table S6. Post-hoc pairwise comparison (multi-group) of alpha diversity indexes. Table S7. Pairwise PERMANOVA analysis of beta diversity. Fig. S1. Bioengineering L. lactis surface expressing CadF protein of C. jejuni. Fig. S2. Standard plot for sodium nitrite (NaNO2) using Griess reagent. Fig. S3. In vitro adhesion assay of uncoated and CS-coated rL. lactis (CadF, Hcp, JlpA) in CEICs. Fig. S4. SDS-PAGE analysis of recombinant CadF, Hcp and JlpA proteins. Fig. S5. sIgA levels in intestinal lavages and fecal soups against individual proteins by ELISA. Fig. S6. Flow cytometric analysis of T-cell subsets in cecal tonsils of immunized birds. Fig. S7. Additional images of tissue sections showing histopathological changes of cecal tissue collected from birds at day 7 post-infection with C. jejuni (TGH 9011). Fig. S8. Clustering heatmap showing relative abundance of microbial genera between treatment groups. [file 40168_2026_2421_MOESM1_ESM.docx]

**Supporting Information**

**Recombinant LAB vector-based multicomponent vaccine against *Campylobacter jejuni* potentially promoting a healthier microbial balance in the poultry gut**

**Prakash Biswas^a^, Sakil Ahmed^a^, Samiran Mondal^b^, Samson Oladokun^c^, Ozan Gundogdu^d^*, and Amirul Islam Mallick^a^***

^a^Department of Biological Sciences, Indian Institute of Science Education and Research Kolkata, Mohanpur, Nadia, 741246, West Bengal, India.

^b^Department of Veterinary Pathology, West Bengal University of Animal and Fishery Sciences, Kolkata, West Bengal 700037, India

^c^Department of Poultry Science, Texas A&M University, College Station, TX 77843, USA

^d^Department of Infection Biology, Faculty of Infectious & Tropical Diseases, London School of Hygiene and Tropical Medicine, London, WC1E 7HT, United Kingdom

***Corresponding Authors**

**Dr. Amirul Islam Mallick, DVM, PhD**

Professor, Department of Biological Sciences, Indian Institute of Science Education and Research Kolkata, Mohanpur, Nadia, West Bengal, 741246, India. Ph. 91-33-61360022-Ext 1221, E-mail: [amallick@iiserkol.ac.in](mailto:amallick@iiserkol.ac.in), ORCID ID: [0000-0002-2265-9856](https://orcid.org/0000-0002-2265-9856)

**Dr. Ozan Gundogdu, PhD**

Associate Professor, Department of Infection Biology, Faculty of Infectious & Tropical Diseases, London School of Hygiene and Tropical Medicine, London, WC1E 7HT, United Kingdom Email: [Ozan.Gundogdu@lshtm.ac.uk](mailto:Ozan.Gundogdu@lshtm.ac.uk), ORCID ID: 0000-0002-3550-0545.

| **Bacterial strains and**  **Plasmids** | **Characteristics** | **Purpose** | **Source** |
| --- | --- | --- | --- |
| **Strains** | | | |
| *E. coli* Top 10 | F- mcrAΔ (mrr-hsdRMS-mcrBC)  Φ80lacZΔM15 Δ lacX74 recA1  araD139 Δ (araleu)7697 galUgalKrpsL  (StrR) endA1 nupG | Recombinant plasmid  storage (*L. lactis-based*  plasmid constructs) | Thermo Fisher  Scientific, USA |
| *E. coli* M15 | F-, Φ80ΔlacM15, thi, lac-, mtl-, recA+, KmR | Recombinant protein expression | [1,2] |
| *E. coli* BL21 | F- ompT gal dcm lon hsdSB(rB-mB-) | Recombinant protein expression | This work |
| *L. lactis* NZ9000 | MG1363 (nisRK genes into  chromosome), Wild type, plasmid-free | Wild-type control | Dr. L. G. Bermúdez  Humarán, INRA, France |
| *L. lactis* NZ9000- JlpA | Cm^r^, NZ9000 harboring pNZ8048-  CWAM6-JlpA | Recombinant protein  expression | [1] |
| *L. lactis* NZ9000- Hcp | Cm^r^, NZ9000 harboring pNZ8048-  CWAM6-Hcp | Recombinant protein  expression | [3] |
| *L. lactis* NZ9000- CadF | Cm^r^, NZ9000 harboring pNZ8048-  CWAM6-CadF | Recombinant protein  expression | This work |
| *C. jejuni* TGH9011 | T6SS-positive *C. jejuni* | *In vivo* challenge study | BEI Resources |
| **Plasmids** | | | |
| pNZ8048-CWA_M6_ | Cm^r^, SPUsp45, M6 cell wall anchor  expressed under the PnisA promoter | *L. lactis-*based recombinant  protein expression vector | Dr. L. G. Bermúdez  Humarán, INRA, France |
| pNZ8048-CWA_M6_-JlpA | Cm^r^, SPUsp45, M6 cell wall anchor  expressed under the PnisA promoter | *L. lactis*-based JlpA protein  expression vector | [1] |
| pNZ8048-CWA_M6_-Hcp | Cm^r^, SPUsp45, M6 cell wall anchor  expressed under the PnisA promoter | *L. lactis*-based Hcp protein  expression vector | [3] |
| pNZ8048-CWA_M6_-CadF | Cm^r^, SPUsp45, M6 cell wall anchor  expressed under the PnisA promoter | *L. lactis*-based CadF protein  expression vector | This work |
| pQE30-JlpA | Amp^r^, pQE30 harbouring JlpA | JlpA protein expression vector | [1] |
| pQE30-Hcp | Amp^r^, pQE30 harbouring Hcp | Hcp protein expression vector | [2] |
| pHisTEV-CadF | Amp^r^, pHisTEV harbouring CadF | CadF protein expression vector | This work |

**Table S1:** The list of bacterial strains and plasmids.

| **Ingredients** | **Pre-starter feed (0 to 10 days)** | **Starter feed (11 to 35 days)** |
| --- | --- | --- |
| Maise | 550 | 590 |
| Soya Deoiled cake* | 390 | 340 |
| Oil | 16.5 | 27 |
| Dicalcium Phosphate | 12 | 12 |
| Line Stone Powder | 16 | 16 |
| Trace Mineral (Manganese, Zinc, Iron, Iodine, Copper, Cobalt) | 2 | 2 |
| Salt | 2.5 | 2.5 |
| Sodium bicarbonate | 1.5 | 1.5 |
| Choline Chloride | 0.5 | 0.5 |
| Lysine | 2.5 | 2 |
| D.L. Methionine | 2.8 | 2.7 |
| Toxin Binder | 1 | 1 |
| Emulsifier | 0.25 | 0.25 |
| Threonine | 0.15 | 0.15 |
| Phytase 5000 | 0.1 | 0.1 |
| Total | 999.9 | 999.8 |

**Table S2:**  Composition of poultry feed used in the present study [4].

| **Antibody name** | **Fluorophore** | **Source** | **Volume utilised** |
| --- | --- | --- | --- |
| Anti-chicken Bu1 | FITC | Southern Biotech, Canada | 0.5 µL |
| Anti-chicken IgA | PE | Southern Biotech, Canada | 0.5 µL |
| Anti-chicken CD3ζ | APC | Southern Biotech, Canada | 0.5 µL |
| Anti-chicken CD4 | FITC | Southern Biotech, Canada | 0.5 µL |
| Anti-chicken TCR γδ | PE | Southern Biotech, Canada | 0.5 µL |
| 7-AAD | Far-red | Southern Biotech, Canada | 1 µL |

**Table S3:**  Detailed list of the antibodies used for flow cytometry.

| **Primers** | **Sequence (5’→3’)** | **References** |
| --- | --- | --- |
| β-actin FP | GAGAAATTGTGCGTGACATCA | [5] |
| β-actin RP | CCTGAACCTCTCATTGCCA |  |
| CXCL8 FP | GGCTTGCTAGGGGAAATGA | [6] |
| CXCL8 RP | AGCTGACTCTGACTAGGAAACTGT |  |
| IL-17 FP | ATTCCAGGTGCGTGAACTCGGC | [2] |
| IL-17 RP | GTGCAGCCCACGGTGATCATTTTC |  |
| IFNγ FP | ACACTGACAAGTCAAAGCCGCACA | [7] |
| IFNγ RP | AGTCGTTCATCGGGAGCTTGGC |  |
| NFκB FP | GAAGGAATCGTACCGGGAACA | [8] |
| NFκB RP | CTCAGAGGGCCTTGTGACAGTAA |  |
| TNFα FP | TTGCAGGCTGTTTCTGCCTCTGC | This work |
| TNFα RP | TGAAGGTGGTGCAGATGGGGC |  |
| IL-1β FP | AGTGAGGCTCAACATTGCGCTGTA | [9] |
| IL-1β RP | TAGAAGATGAAGCGGGTCAGCTCG |  |
| iNOS FP | GAACAGCCAGCTCATCCGATA | This work |
| iNOS RP | CCCAAGCTCAATGCACAACTT |  |
| CadF FP | CGGCGCGCATGCGCTGATAACAATGTAAAATTTG | This work |
| CadF RP | CGCGCGGCTAGCGATCTTAAAATAAATTTAGCATCC |  |

**Table S4:**  Cytokine gene primers used for qRT-PCR and CadF cloning primers.


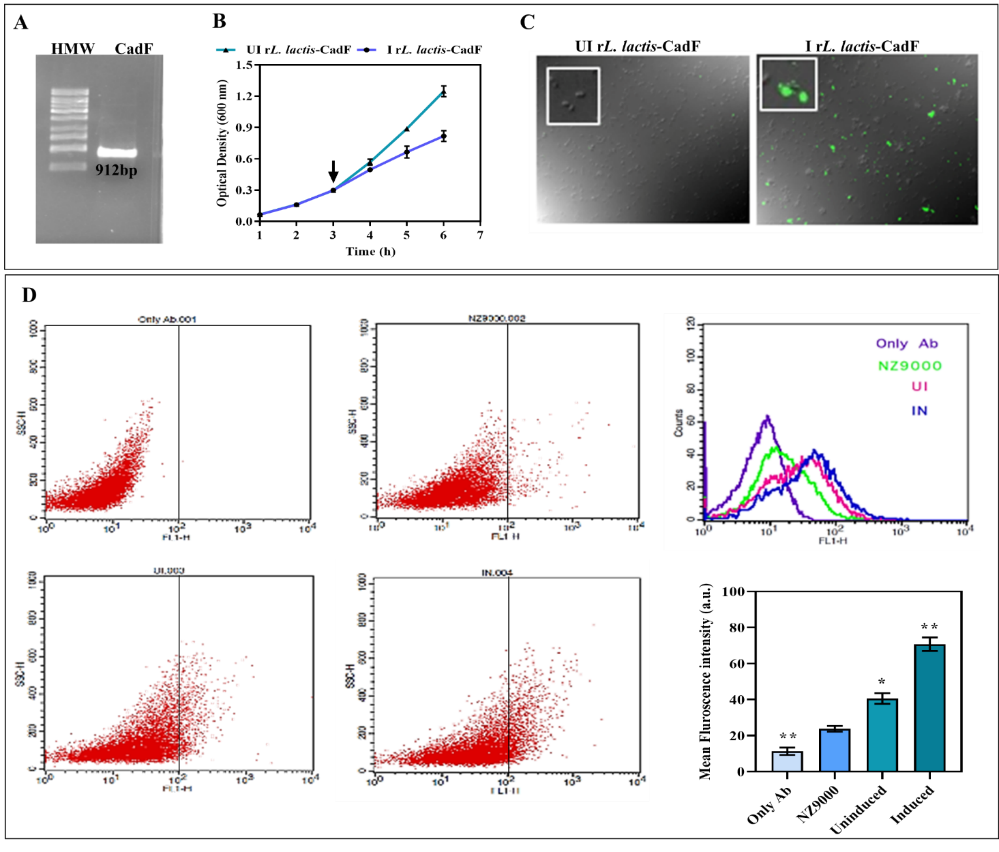


**Figure S1:** **Bioengineering *L*. *lactis* surface expressing CadF protein of *C. jejuni****.* The gene encoding the truncated CadF sequence was successfully cloned into the nisin-inducible *Lactococcal* expression vector pNZ8048-CWA_M6_, positioned between the N-terminal signal peptide (SP) of USP_45_ and the C-terminal CWA_M6_ domain. Restriction digestion of recombinant plasmid (pNZ8048-USP_45_**-**CadF-CWA_M6_) with *Sph*I/*Nhe*I endonucleases and gene sequencing confirmed the successful cloning of the desired gene. (**A)** *L. lactis* NZ9000 cells were electro-transformed with the pNZ-CadF plasmid, and the positive transformants were screened using chloramphenicol as a selection marker and gene amplification (~912 bp) using a specific primer set. (**B)** *In vitro* growth kinetics of uninduced and nisin-induced r*L. lactis* cells. Cells were induced with Nisin (Sigma, USA) (15 ng/mL) when OD_600_ reached ~0.2-0.3. **(C)** Further indirect immunofluorescent assay and (**D**) flow cytometric analysis of nisin-induced r*L. lactis* cells confirm optimal surface expression of CadF protein.

**Figure S2:** Standard plot for sodium nitrite (NaNO_2_) using Griess reagent. This plot was used to quantify NO production (refer to Figure 2D).

*
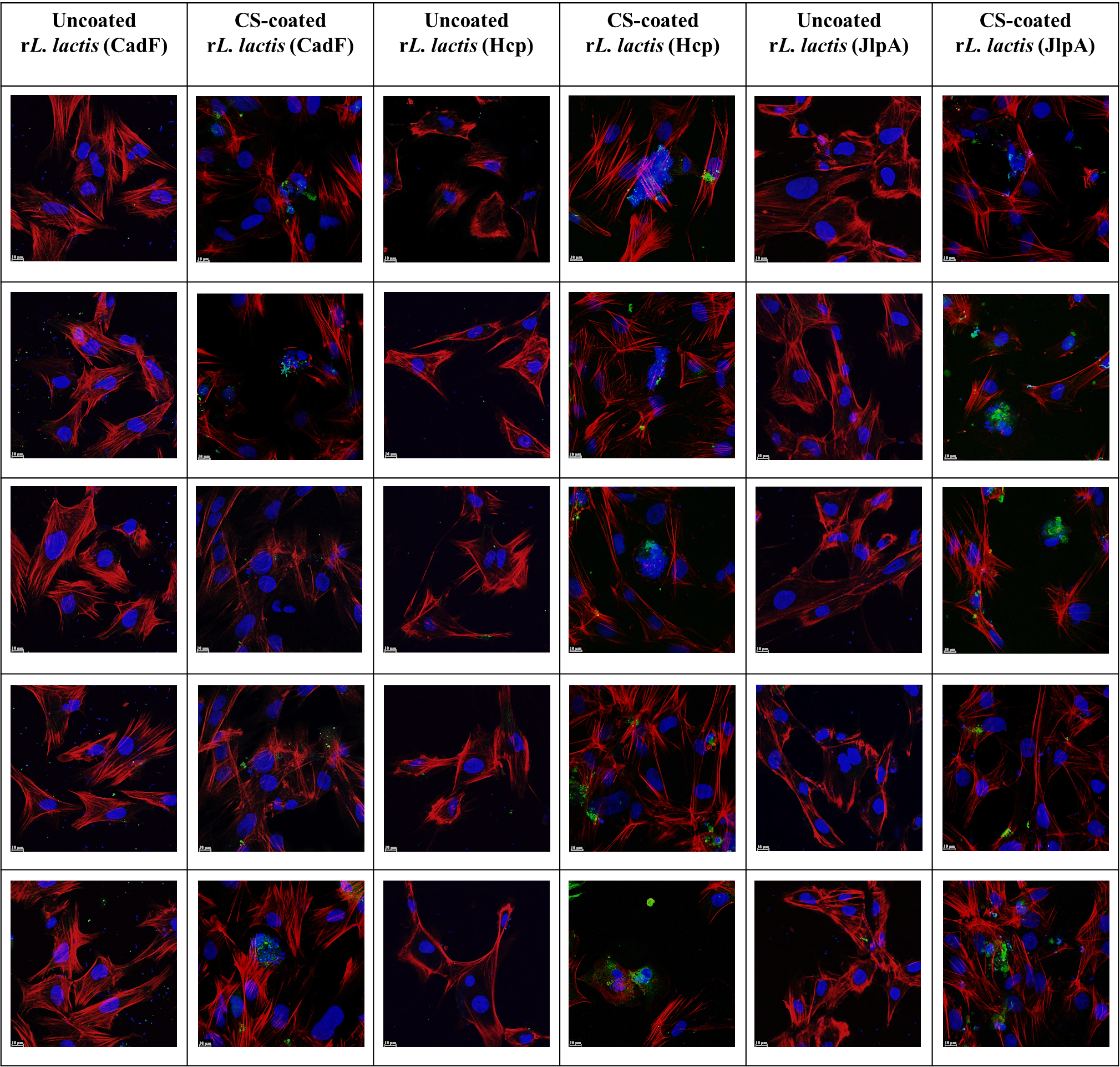
*

**Figure S3:** ***In vitro* adhesion assay of uncoated and CS-coated r*L. Lactis* (CadF, Hcp, JlpA) in CEICs.** The cells were incubated with induced uncoated and CS-coated r*L. lactis* 4 h at 37 °C with 5% CO_2_, at a ratio of cell vs bacteria 1:1000. CLSM images of cells incubated with CS-coated r*L. lactis* cells showed enhanced cell adhesion compared to uncoated bacteria. Scale bar: 10 μm.


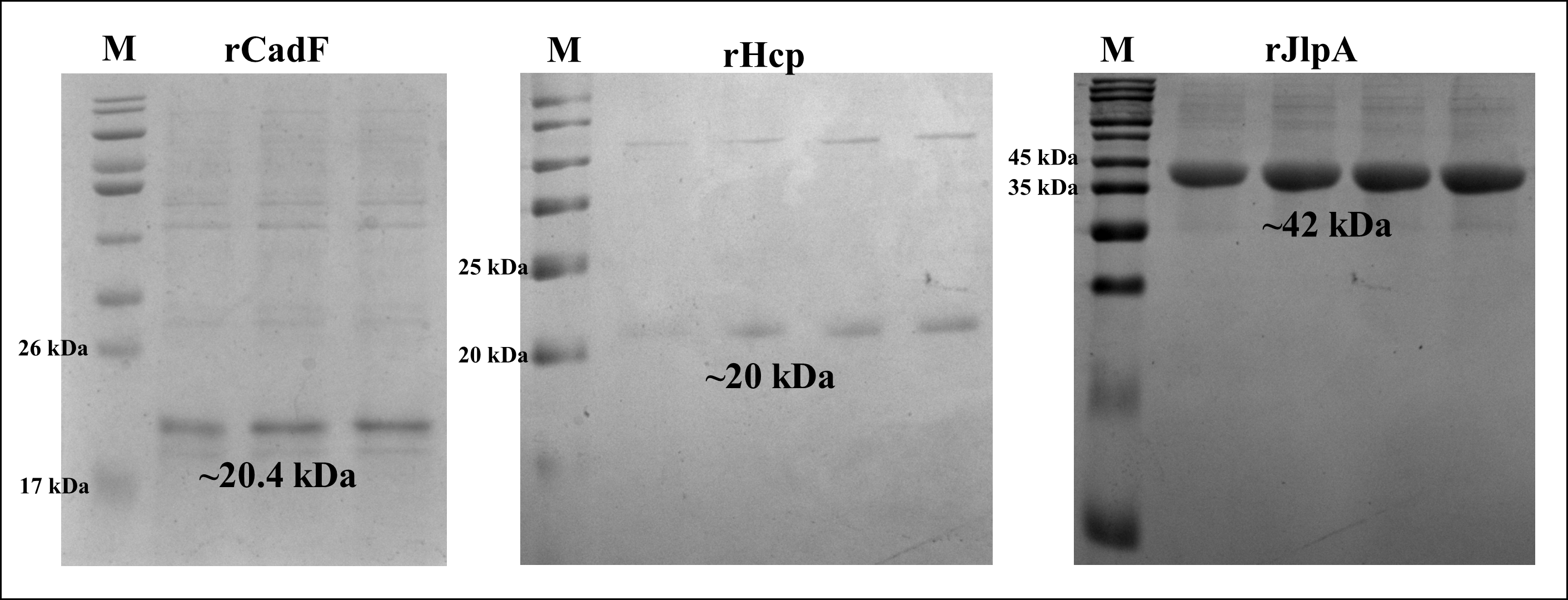


**Figure S4: SDS-PAGE analysis of recombinant CadF, Hcp, and JlpA proteins.** Representative Coomassie-stained SDS-polyacrylamide gels showing protein bands at the expected molecular weights. Left panel: SDS-PAGE showing a prominent band at ~20.4 kDa for CadF protein. Middle panel: SDS-PAGE indicating protein bands migrating at approximately ~20 kDa for the Hcp protein. Right panel: SDS-PAGE showing a strong band at ~42 kDa for JlpA protein. The positions of the target proteins are indicated with approximate molecular weights.


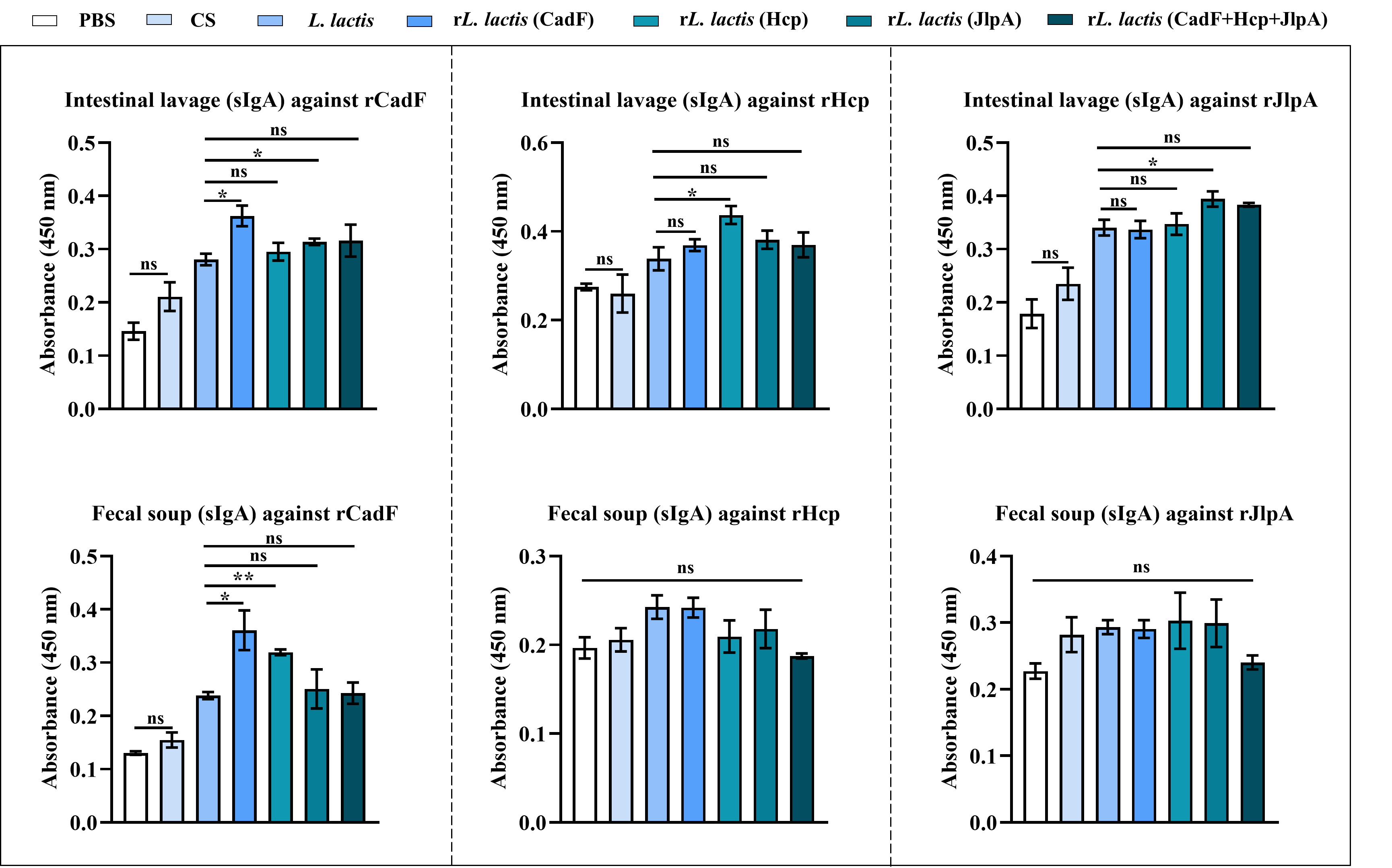


**Figure S5:** Antigen-specific mucosal sIgA responses in intestinal lavage and fecal soup samples following oral immunization with recombinant *L. lactis* strains. ELISA plates were coated individually with purified recombinant CadF, Hcp, or JlpA proteins, and sIgA levels were measured in intestinal lavage and fecal soup samples collected from experimental birds. Antibody responses in birds immunized with r*L. lactis* expressing CadF, Hcp, or JlpA were compared with those in the *L. lactis* control group. Each bar represents the mean absorbance (A450) ± SE of 12 birds pooled in 4 replicates from two independent experiments. Asterisks indicate a statistically significant difference (**p* < 0.05) compared to the control group (PBS).


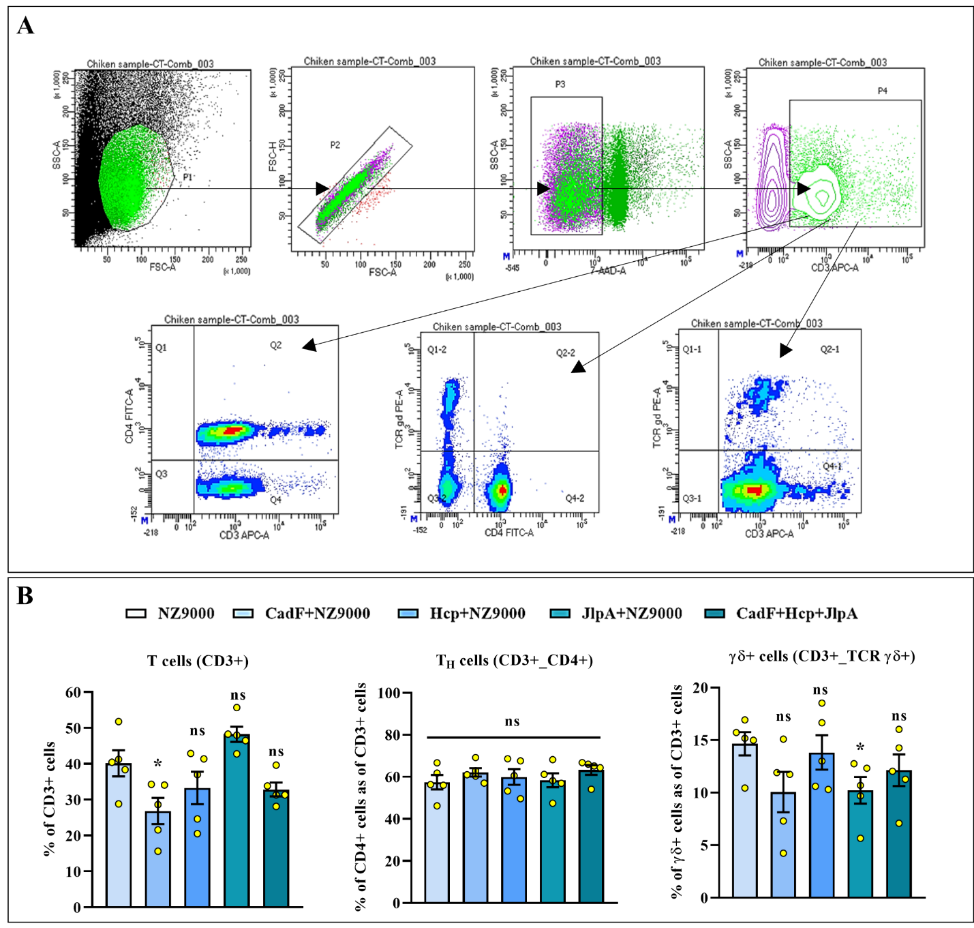


**Figure S6: Flow cytometric analysis of T-cell subsets in cecal tonsils of immunized birds.** Cecal tonsils were collected from birds at day 28 post-first immunization and processed to isolate mononuclear cells. **(A)** Panel showing the gating strategy for T cells present in the cecal tonsil (CT) of experimental birds. **(B)** Flow cytometric analysis of T cells suggests no changes in the CD3^+^, CD4^+,^ and TCRγδ^+^ populations in birds administered with r*L. lactis* (CadF, JlpA, and Hcp) compared to other experimental groups.


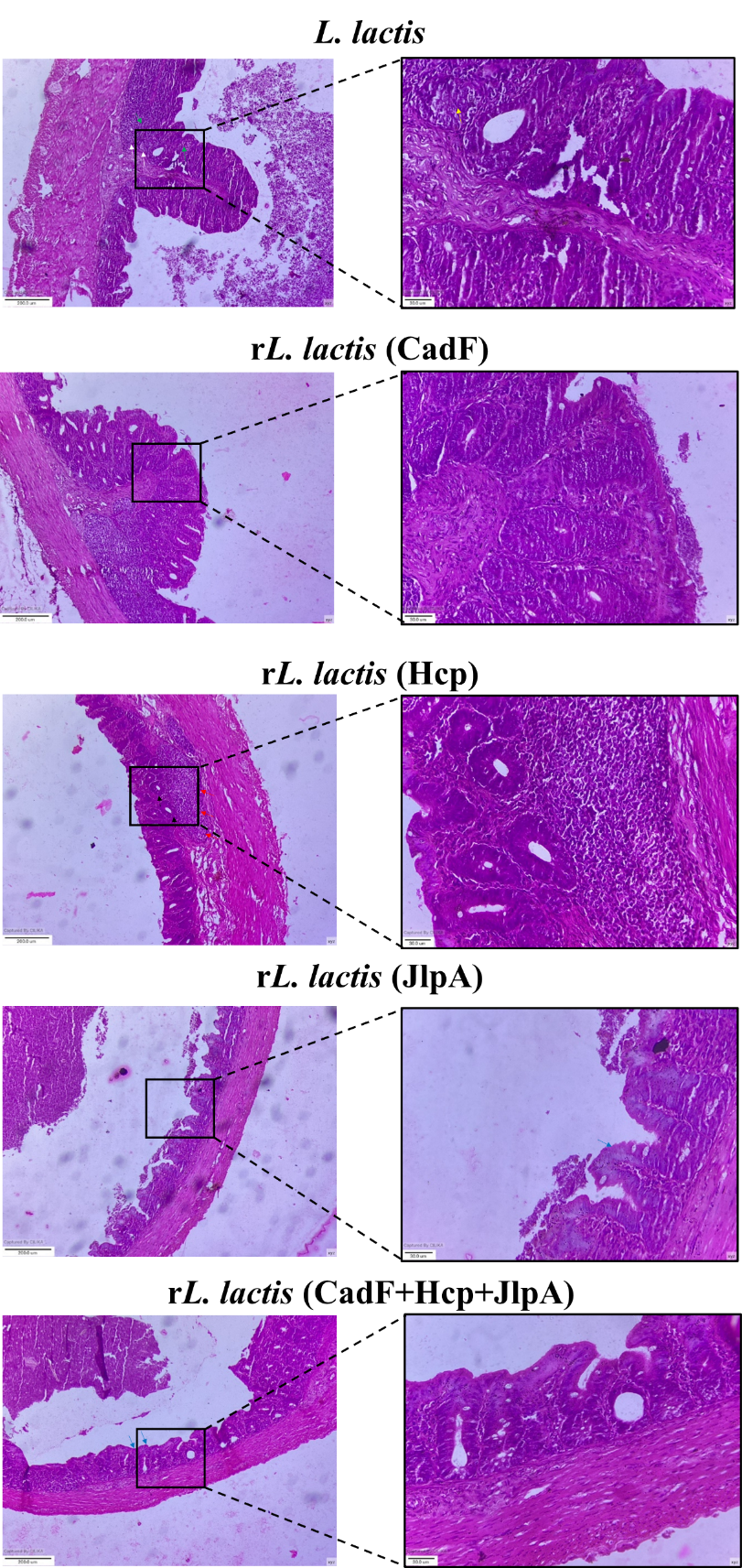


**Figure S7:** **Additional images of tissue sections showing histopathological changes** of cecal tissue collected from birds at day 7 post-infection with *C. jejuni* (TGH 9011). Green arrow: cellular infiltration, yellow arrow: crypt disruption, white arrow: tissue necrosis, red arrow: lymphoid accumulation, black arrow: well-organized crypt, blue arrow: intact epithelia.

| **PBS vs Baseline** | | | | | | | | | | | | | |
| --- | --- | --- | --- | --- | --- | --- | --- | --- | --- | --- | --- | --- | --- |
| **Phylum** | **Log2FC** | **St. Error** | | | | **P-value** | | | | **FDR** | | | |
| Armatimonadota | 0.909 | 0.312 | | | | 0.00351 | | | | 0.0181 | | | |
| Actinomycetota | 0.935 | 0.477 | | | | 0.0499 | | | | 0.132 | | | |
| Candidatus_Bipolaricaulota | 0.988 | 0.568 | | | | 0.0818 | | | | 0.176 | | | |
| Aquificota | 1.41 | 0.895 | | | | 0.115 | | | | 0.229 | | | |
| Acidobacteriota | -0.584 | 0.395 | | | | 0.139 | | | | 0.26 | | | |
| Fusobacteriota | 0.898 | 0.725 | | | | 0.216 | | | | 0.345 | | | |
| Nitrospirota | -0.802 | 0.658 | | | | 0.223 | | | | 0.345 | | | |
| Thermodesulfobacteriota | 0.872 | 0.838 | | | | 0.298 | | | | 0.437 | | | |
| Bacteroidota | 0.778 | 0.772 | | | | 0.314 | | | | 0.445 | | | |
| Campylobacterota | -0.417 | 0.632 | | | | 0.51 | | | | 0.632 | | | |
| Candidatus_Omnitrophota | 0.0354 | 0.704 | | | | 0.96 | | | | 0.98 | | | |
| **PBS vs UU** | | | | | | | | | | | | | |
| **Phylum** | **Log_2_FC** | | **St. Error** | | | | **P-value** | | | | **FDR** | | |
| Armatimonadota | 1.37 | | 0.316 | | | | 1.45E-5 | | | | 9.83E-5 | | |
| Candidatus_Bipolaricaulota | 1.49 | | 0.427 | | | | 4.75E-4 | | | | 0.0019 | | |
| Fusobacteriota | 1.23 | | 0.541 | | | | 0.0226 | | | | 0.0552 | | |
| Actinomycetota | 0.908 | | 0.477 | | | | 0.0568 | | | | 0.119 | | |
| Aquificota | 1.23 | | 0.76 | | | | 0.105 | | | | 0.18 | | |
| Bacteroidota | 0.885 | | 0.646 | | | | 0.17 | | | | 0.268 | | |
| Acidobacteriota | -0.309 | | 0.397 | | | | 0.436 | | | | 0.582 | | |
| Campylobacterota | -0.36 | | 0.516 | | | | 0.486 | | | | 0.602 | | |
| Thermodesulfobacteriota | 0.428 | | 0.603 | | | | 0.478 | | | | 0.602 | | |
| Candidatus_Omnitrophota | 0.15 | | 0.578 | | | | 0.796 | | | | 0.891 | | |
| Nitrospirota | -2.22E-7 | | 0.82 | | | | 1.0 | | | | 1.0 | | |
| **PBS vs CS** | | | | | | | | | | | | | |
| **Phylum** | **Log2FC** | | | | **St. Error** | | | | **P-value** | | | **FDR** | |
| Candidatus_Bipolaricaulota | 0.511 | | | | 0.39 | | | | 0.19 | | | 0.315 | |
| Armatimonadota | 0.376 | | | | 0.308 | | | | 0.222 | | | 0.345 | |
| Thermodesulfobacteriota | -0.625 | | | | 0.647 | | | | 0.334 | | | 0.459 | |
| Fusobacteriota | 0.396 | | | | 0.528 | | | | 0.453 | | | 0.595 | |
| Actinomycetota | 0.354 | | | | 0.508 | | | | 0.486 | | | 0.603 | |
| Aquificota | 0.333 | | | | 0.747 | | | | 0.656 | | | 0.769 | |
| Candidatus_Omnitrophota | 0.279 | | | | 0.719 | | | | 0.698 | | | 0.797 | |
| Acidobacteriota | -0.114 | | | | 0.399 | | | | 0.775 | | | 0.853 | |
| Nitrospirota | 0.185 | | | | 0.832 | | | | 0.824 | | | 0.873 | |
| Bacteroidota | 0.13 | | | | 0.69 | | | | 0.851 | | | 0.888 | |
| Campylobacterota | 0.00662 | | | | 0.516 | | | | 0.99 | | | 0.99 | |
| **PBS vs *L. lactis*** | | | | | | | | | | | | | |
| **Phylum** | **Log2FC** | | | **St. Error** | | | | **P-value** | | | | | **FDR** |
| Armatimonadota | 2.15 | | | 0.361 | | | | 2.44E-9 | | | | | 7.15E-8 |
| Actinomycetota | -2.35 | | | 0.474 | | | | 6.82E-7 | | | | | 1.17E-5 |
| Acidobacteriota | -1.35 | | | 0.392 | | | | 5.52E-4 | | | | | 0.00343 |
| Candidatus_Omnitrophota | -1.66 | | | 0.482 | | | | 5.85E-4 | | | | | 0.00343 |
| Thermodesulfobacteriota | -1.77 | | | 0.559 | | | | 0.00155 | | | | | 0.00719 |
| Bacteroidota | -1.42 | | | 0.645 | | | | 0.0274 | | | | | 0.0755 |
| Aquificota | -1.46 | | | 0.677 | | | | 0.0312 | | | | | 0.0824 |
| Candidatus_Bipolaricaulota | 0.969 | | | 0.459 | | | | 0.0346 | | | | | 0.0824 |
| Nitrospirota | -1.34 | | | 0.636 | | | | 0.0356 | | | | | 0.0824 |
| Fusobacteriota | -0.962 | | | 0.494 | | | | 0.0516 | | | | | 0.106 |
| Campylobacterota | 0.257 | | | 0.517 | | | | 0.619 | | | | | 0.718 |
| **PBS vs r*L. lactis* (CadF)** | | | | | | | | | | | | | |
| **Phylum** | **Log2FC** | | | **St. Error** | | | | **P-value** | | | | | **FDR** |
| Actinomycetota | -2.22 | | | 0.474 | | | | 2.74E-6 | | | | | 3.45E-5 |
| Armatimonadota | 2.13 | | | 0.543 | | | | 8.75E-5 | | | | | 7.0E-4 |
| Thermodesulfobacteriota | -2.25 | | | 0.587 | | | | 1.26E-4 | | | | | 7.94E-4 |
| Bacteroidota | -2.36 | | | 0.645 | | | | 2.54E-4 | | | | | 0.0014 |
| Candidatus_Omnitrophota | -1.73 | | | 0.481 | | | | 3.2E-4 | | | | | 0.00166 |
| Acidobacteriota | -1.32 | | | 0.392 | | | | 7.45E-4 | | | | | 0.00345 |
| Candidatus_Bipolaricaulota | 1.34 | | | 0.423 | | | | 0.00155 | | | | | 0.0062 |
| Nitrospirota | -1.57 | | | 0.652 | | | | 0.0159 | | | | | 0.0423 |
| Aquificota | -1.61 | | | 0.676 | | | | 0.0175 | | | | | 0.0454 |
| Fusobacteriota | -0.399 | | | 0.496 | | | | 0.421 | | | | | 0.562 |
| Campylobacterota | 0.147 | | | 0.516 | | | | 0.776 | | | | | 0.853 |
| **PBS vs r*L. lactis* (Hcp)** | | | | | | | | | | | | | |
| **Phylum** | **Log2FC** | | | **St. Error** | | | | **P-value** | | | | | **FDR** |
| Armatimonadota | 2.35 | | | 0.559 | | | | 2.53E-5 | | | | | 5.56E-4 |
| Actinomycetota | -1.84 | | | 0.474 | | | | 1.02E-4 | | | | | 0.0018 |
| Candidatus_Bipolaricaulota | 1.34 | | | 0.581 | | | | 0.0212 | | | | | 0.103 |
| Bacteroidota | -1.35 | | | 0.645 | | | | 0.0367 | | | | | 0.154 |
| Campylobacterota | 1.03 | | | 0.518 | | | | 0.0465 | | | | | 0.173 |
| Candidatus_Omnitrophota | -0.762 | | | 0.486 | | | | 0.117 | | | | | 0.258 |
| Acidobacteriota | -0.594 | | | 0.395 | | | | 0.133 | | | | | 0.276 |
| Aquificota | -0.784 | | | 0.677 | | | | 0.247 | | | | | 0.419 |
| Thermodesulfobacteriota | -0.695 | | | 0.646 | | | | 0.282 | | | | | 0.469 |
| Nitrospirota | -0.717 | | | 0.694 | | | | 0.302 | | | | | 0.483 |
| Fusobacteriota | -0.19 | | | 0.497 | | | | 0.702 | | | | | 0.853 |
| **PBS vs r*L. lactis* (JlpA)** | | | | | | | | | | | | | |
| **Phylum** | **Log2FC** | | | **St. Error** | | | | **P-value** | | | | | **FDR** |
| Actinomycetota | -2.08 | | | 0.474 | | | | 1.13E-5 | | | | | 2.5E-4 |
| Armatimonadota | 2.35 | | | 0.559 | | | | 2.53E-5 | | | | | 3.7E-4 |
| Candidatus_Bipolaricaulota | 1.8 | | | 0.485 | | | | 2.03E-4 | | | | | 0.00255 |
| Acidobacteriota | -1.27 | | | 0.392 | | | | 0.00123 | | | | | 0.009 |
| Candidatus_Omnitrophota | -1.27 | | | 0.483 | | | | 0.00833 | | | | | 0.0407 |
| Bacteroidota | -1.13 | | | 0.645 | | | | 0.0795 | | | | | 0.2 |
| Aquificota | -1.07 | | | 0.677 | | | | 0.113 | | | | | 0.256 |
| Nitrospirota | -0.909 | | | 0.657 | | | | 0.166 | | | | | 0.333 |
| Thermodesulfobacteriota | -0.681 | | | 0.562 | | | | 0.226 | | | | | 0.406 |
| Campylobacterota | 0.551 | | | 0.517 | | | | 0.287 | | | | | 0.486 |
| Fusobacteriota | -0.116 | | | 0.497 | | | | 0.816 | | | | | 0.872 |
| **PBS vs r*L. lactis* (CadF+Hcp+JlpA)** | | | | | | | | | | | | | |
| **Phylum** | **Log2FC** | | | **St. Error** | | | | **P-value** | | | | | **FDR** |
| Armatimonadota | 1.87 | | | 0.404 | | | | 3.77E-6 | | | | | 8.3E-5 |
| Actinomycetota | -1.75 | | | 0.474 | | | | 2.29E-4 | | | | | 0.00227 |
| Bacteroidota | -2.21 | | | 0.645 | | | | 6.07E-4 | | | | | 0.00486 |
| Candidatus_Bipolaricaulota | 1.51 | | | 0.59 | | | | 0.0104 | | | | | 0.0611 |
| Candidatus_Omnitrophota | -1.14 | | | 0.484 | | | | 0.0184 | | | | | 0.0871 |
| Campylobacterota | 0.828 | | | 0.518 | | | | 0.109 | | | | | 0.254 |
| Nitrospirota | -1.07 | | | 0.655 | | | | 0.101 | | | | | 0.254 |
| Aquificota | -0.937 | | | 0.677 | | | | 0.166 | | | | | 0.341 |
| Thermodesulfobacteriota | -0.838 | | | 0.645 | | | | 0.194 | | | | | 0.363 |
| Acidobacteriota | -0.417 | | | 0.427 | | | | 0.329 | | | | | 0.527 |
| Fusobacteriota | 0.195 | | | 0.526 | | | | 0.711 | | | | | 0.811 |

**Table S5: Multiple linear regression with covariate adjustment of cecal microbiota at the phylum level.** The MicrobiomeAnalyst (2.0), a freely available software, was used to perform multiple regression analyses across experimental groups. The tool uses MaAsLin2 to fit general models to identify associations between microbial features and experimental metadata. A Zero-Inflated Negative Binomial Model (ZINB) was fitted to each microbial feature, including the primary metadata and covariates.

| **Chao1** | | | |
| --- | --- | --- | --- |
| **Pair** | **Statistic** | **P-value** | **FDR** |
| Baseline vs CS | -0.7387 | 0.49235 | 0.68172 |
| Baseline vs L._lactis | -5.626 | 0.00524 | 0.063954 |
| Baseline vs PBS | -0.85651 | 0.42462 | 0.65097 |
| Baseline vs rL._lactis_(CadF) | -4.2464 | 0.016894 | 0.070517 |
| Baseline vs rL._lactis_(CadF+Hcp+JlpA) | -2.1524 | 0.11347 | 0.24029 |
| Baseline vs rL._lactis_(Hcp) | -3.608 | 0.027017 | 0.08105 |
| Baseline vs rL._lactis_(JlpA) | -5.0298 | 0.0078753 | 0.063954 |
| Baseline vs UU | -2.07 | 0.12173 | 0.24346 |
| CS vs L._lactis | -4.7123 | 0.0054202 | 0.063954 |
| CS vs rL._lactis_(CadF) | -3.6419 | 0.019588 | 0.070517 |
| CS vs rL._lactis_(CadF+Hcp+JlpA) | -1.8312 | 0.1492 | 0.26856 |
| CS vs rL._lactis_(Hcp) | -3.0144 | 0.0353 | 0.088312 |
| CS vs rL._lactis_(JlpA) | -4.1756 | 0.0090239 | 0.063954 |
| L._lactis vs rL._lactis_(CadF) | 0.19805 | 0.84994 | 0.89993 |
| L._lactis vs rL._lactis_(CadF+Hcp+JlpA) | 0.8314 | 0.44759 | 0.65097 |
| L._lactis vs rL._lactis_(Hcp) | 0.84185 | 0.43356 | 0.65097 |
| L._lactis vs rL._lactis_(JlpA) | 0.4117 | 0.69486 | 0.78172 |
| PBS vs CS | -0.069136 | 0.94745 | 0.94745 |
| PBS vs L._lactis | -5.1564 | 0.0068991 | 0.063954 |
| PBS vs rL._lactis_(CadF) | -3.8779 | 0.021911 | 0.071709 |
| **Shannon** | | | |
| **Pair** | **Statistic** | **P-value** | **FDR** |
| Baseline vs CS | -0.94281 | 0.39154 | 0.55982 |
| Baseline vs L._lactis | -5.0328 | 0.0029105 | 0.10478 |
| Baseline vs PBS | -1.2612 | 0.25562 | 0.48432 |
| Baseline vs rL._lactis_(CadF) | -3.9041 | 0.013171 | 0.13368 |
| Baseline vs rL._lactis_(CadF+Hcp+JlpA) | -0.40903 | 0.70591 | 0.79415 |
| Baseline vs rL._lactis_(Hcp) | 0.57017 | 0.59842 | 0.75296 |
| Baseline vs rL._lactis_(JlpA) | -1.8216 | 0.15245 | 0.36589 |
| Baseline vs UU | -2.4617 | 0.053838 | 0.21535 |
| CS vs L._lactis | -2.2615 | 0.085756 | 0.28066 |
| CS vs rL._lactis_(CadF) | -2.4349 | 0.050854 | 0.21535 |
| CS vs rL._lactis_(CadF+Hcp+JlpA) | 0.14355 | 0.89187 | 0.94433 |
| CS vs rL._lactis_(Hcp) | 1.1531 | 0.29532 | 0.48478 |
| CS vs rL._lactis_(JlpA) | -1.1798 | 0.29625 | 0.48478 |
| L._lactis vs rL._lactis_(CadF) | -0.93175 | 0.40431 | 0.55982 |
| L._lactis vs rL._lactis_(CadF+Hcp+JlpA) | 1.4692 | 0.22955 | 0.48432 |
| L._lactis vs rL._lactis_(Hcp) | 3.1932 | 0.037812 | 0.19446 |
| L._lactis vs rL._lactis_(JlpA) | -0.043294 | 0.96796 | 0.96796 |
| PBS vs CS | -0.14865 | 0.88865 | 0.94433 |
| PBS vs L._lactis | -4.171 | 0.0059699 | 0.10746 |
| PBS vs rL._lactis_(CadF) | -3.241 | 0.029819 | 0.17892 |
| **Simpson** | | | |
| **Pair** | **Statistic** | **P-value** | **FDR** |
| Baseline vs CS | 0.39635 | 0.71173 | 0.83121 |
| Baseline vs L._lactis | 0.15848 | 0.87958 | 0.90472 |
| Baseline vs PBS | -0.30815 | 0.76847 | 0.84763 |
| Baseline vs rL._lactis_(CadF) | -0.83054 | 0.45068 | 0.7736 |
| Baseline vs rL._lactis_(CadF+Hcp+JlpA) | 1.6023 | 0.20249 | 0.50945 |
| Baseline vs rL._lactis_(Hcp) | 2.9041 | 0.056848 | 0.37776 |
| Baseline vs rL._lactis_(JlpA) | 0.44072 | 0.68794 | 0.83121 |
| Baseline vs UU | -1.8139 | 0.12455 | 0.50945 |
| CS vs L._lactis | -0.28009 | 0.79135 | 0.84763 |
| CS vs rL._lactis_(CadF) | -0.93959 | 0.38385 | 0.76771 |
| CS vs rL._lactis_(CadF+Hcp+JlpA) | 1.3536 | 0.2488 | 0.55434 |
| CS vs rL._lactis_(Hcp) | 2.509 | 0.06296 | 0.37776 |
| CS vs rL._lactis_(JlpA) | 0.27097 | 0.80054 | 0.84763 |
| L._lactis vs rL._lactis_(CadF) | -0.88875 | 0.41581 | 0.7736 |
| L._lactis vs rL._lactis_(CadF+Hcp+JlpA) | 1.5448 | 0.21227 | 0.50945 |
| L._lactis vs rL._lactis_(Hcp) | 2.819 | 0.057987 | 0.37776 |
| L._lactis vs rL._lactis_(JlpA) | 0.39742 | 0.71576 | 0.83121 |
| PBS vs CS | 0.56873 | 0.60067 | 0.83121 |
| PBS vs L._lactis | 0.42796 | 0.6852 | 0.83121 |
| PBS vs rL._lactis_(CadF) | -0.666 | 0.54163 | 0.77995 |
| **Fisher** | | | |
| **Pair** | **Statistic** | **P-value** | **FDR** |
| Baseline vs CS | -1.0486 | 0.33809 | 0.50714 |
| Baseline vs L._lactis | -10.669 | 5.64E-05 | 0.0017278 |
| Baseline vs PBS | -0.88543 | 0.41056 | 0.52787 |
| Baseline vs rL._lactis_(CadF) | -6.904 | 0.0019106 | 0.0085976 |
| Baseline vs rL._lactis_(CadF+Hcp+JlpA) | -3.3324 | 0.037117 | 0.078601 |
| Baseline vs rL._lactis_(Hcp) | -5.5254 | 0.004818 | 0.014454 |
| Baseline vs rL._lactis_(JlpA) | -8.4591 | 3.99E-04 | 0.0035932 |
| Baseline vs UU | -2.6778 | 0.068848 | 0.12232 |
| CS vs L._lactis | -8.3354 | 1.66E-04 | 0.0019866 |
| CS vs rL._lactis_(CadF) | -5.7642 | 0.002185 | 0.00874 |
| CS vs rL._lactis_(CadF+Hcp+JlpA) | -2.8192 | 0.052025 | 0.098575 |
| CS vs rL._lactis_(Hcp) | -4.5203 | 0.0066387 | 0.018273 |
| CS vs rL._lactis_(JlpA) | -6.8158 | 5.71E-04 | 0.0041119 |
| L._lactis vs rL._lactis_(CadF) | 0.26715 | 0.8004 | 0.84748 |
| L._lactis vs rL._lactis_(CadF+Hcp+JlpA) | 0.95317 | 0.39949 | 0.52787 |
| L._lactis vs rL._lactis_(Hcp) | 1.3399 | 0.24143 | 0.37789 |
| L._lactis vs rL._lactis_(JlpA) | 0.44972 | 0.6697 | 0.75341 |
| PBS vs CS | -0.36446 | 0.73003 | 0.7964 |
| PBS vs L._lactis | -10.413 | 9.60E-05 | 0.0017278 |
| PBS vs rL._lactis_(CadF) | -6.5465 | 0.002954 | 0.010634 |

**Table S6:** The table summarises the post-hoc pairwise comparison (multi-group) of alpha diversity indexes (Chao1, Shannon, Simpson, and Fisher). Regular Welch t-tests/ANOVA were performed for each pair. The multi-testing adjustment is based on the Benjamini-Hochberg procedure (FDR).

| **Beta diversity (PERMANOVA)** | | | | |
| --- | --- | --- | --- | --- |
| **Pair** | **F-value** | **R-squared** | **P-value** | **FDR** |
| Baseline vs UU | 6.1114 | 0.5046 | 0.027 | 0.054 |
| Baseline vs PBS | 6.046 | 0.50191 | 0.023 | 0.054 |
| Baseline vs CS | 4.3581 | 0.42074 | 0.028 | 0.054 |
| Baseline vs L._lactis | 13.69 | 0.69528 | 0.027 | 0.054 |
| Baseline vs rL._lactis_(CadF) | 11.493 | 0.65701 | 0.026 | 0.054 |
| Baseline vs rL._lactis_(Hcp) | 24.972 | 0.80628 | 0.04 | 0.054667 |
| Baseline vs rL._lactis_(JlpA) | 8.5266 | 0.58696 | 0.029 | 0.054 |
| Baseline vs rL._lactis_(CadF+Hcp+JlpA) | 14.084 | 0.70126 | 0.028 | 0.054 |
| UU vs PBS | 4.8562 | 0.44732 | 0.024 | 0.054 |
| UU vs CS | 3.329 | 0.35684 | 0.041 | 0.054667 |
| UU vs L._lactis | 20.316 | 0.772 | 0.031 | 0.054 |
| UU vs rL._lactis_(CadF) | 14.459 | 0.70673 | 0.029 | 0.054 |
| UU vs rL._lactis_(Hcp) | 35.516 | 0.85548 | 0.018 | 0.054 |
| UU vs rL._lactis_(JlpA) | 9.6979 | 0.61778 | 0.028 | 0.054 |
| UU vs rL._lactis_(CadF+Hcp+JlpA) | 19.215 | 0.76205 | 0.027 | 0.054 |
| PBS vs CS | 1.632 | 0.21383 | 0.112 | 0.13006 |
| PBS vs L._lactis | 20.215 | 0.77113 | 0.033 | 0.054 |
| PBS vs rL._lactis_(CadF) | 13.709 | 0.69556 | 0.033 | 0.054 |
| PBS vs rL._lactis_(Hcp) | 38.027 | 0.86372 | 0.038 | 0.054667 |
| PBS vs rL._lactis_(JlpA) | 10.165 | 0.62884 | 0.027 | 0.054 |

**Table S7:** The table summarises the results of the pairwise PERMANOVA analysis of beta diversity. The multi-testing adjustment is based on the Benjamini-Hochberg procedure (FDR).


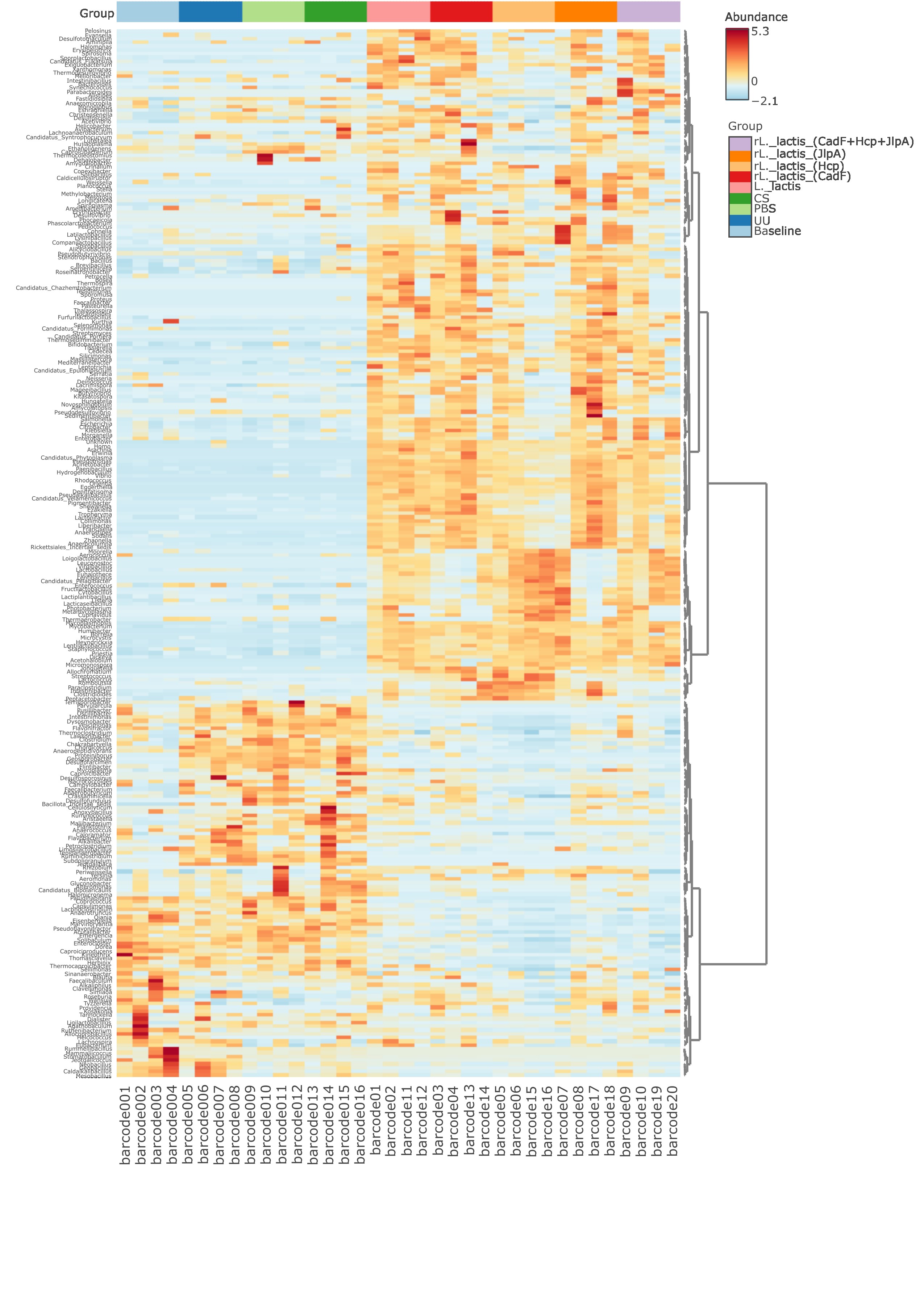
**Figure S8:** The clustering heatmap showed the relative abundance of microbial genera between treatment groups.

**References:**

1. Gorain C, Singh A, Bhattacharyya S, Kundu A, Lahiri A, Gupta S, et al. Mucosal delivery of live Lactococcus lactis expressing functionally active JlpA antigen induces potent local immune response and prevent enteric colonization of Campylobacter jejuni in chickens. Vaccine. 2020;38:1630–42. https://doi.org/10.1016/j.vaccine.2019.12.064

2. Singh A, Nisaa K, Bhattacharyya S, Mallick AI. Immunogenicity and protective efficacy of mucosal delivery of recombinant hcp of Campylobacter jejuni Type VI secretion system (T6SS) in chickens. Mol Immunol. 2019;111:182–97. https://doi.org/10.1016/j.molimm.2019.04.016

3. Gorain C, Khan A, Singh A, Mondal S, Mallick AI. Bioengineering of LAB vector expressing Haemolysin co-regulated protein (Hcp): a strategic approach to control gut colonization of Campylobacter jejuni in a murine model. Gut Pathogens. 2021;13:48. https://doi.org/10.1186/s13099-021-00444-2

4. Jayaraman S, Das PP, Saini PC, Roy B, Chatterjee PN. Use of *Bacillus Subtilis* PB6 as a potential antibiotic growth promoter replacement in improving performance of broiler birds. Poultry Science. 2017;96:2614–22. https://doi.org/10.3382/ps/pex079

5. Li Y-P, Ingmer H, Madsen M, Bang DD. Cytokine responses in primary chicken embryo intestinal cells infected with Campylobacter jejuni strains of human and chicken origin and the expression of bacterial virulence-associated genes. BMC Microbiol. 2008;8:107. https://doi.org/10.1186/1471-2180-8-107

6. Hong YH, Lillehoj HS, Lee SH, Dalloul RA, Lillehoj EP. Analysis of chicken cytokine and chemokine gene expression following Eimeria acervulina and Eimeria tenella infections. Vet Immunol Immunopathol. 2006;114:209–23. https://doi.org/10.1016/j.vetimm.2006.07.007

7. Mallick AI, Haq K, Brisbin JT, Mian MF, Kulkarni RR, Sharif S. Assessment of bioactivity of a recombinant chicken interferon-gamma expressed using a baculovirus expression system. J Interferon Cytokine Res. 2011;31:493–500. https://doi.org/10.1089/jir.2010.0130

8. Chiang H-I, Berghman LR, Zhou H. Inhibition of NF-kB 1 (NF-kBp50) by RNA interference in chicken macrophage HD11 cell line challenged with Salmonellaenteritidis. Genet Mol Biol. 2009;32:507–15. https://doi.org/10.1590/S1415-47572009000300013

9. Bhowmick S, Gupta S, Mondal S, Mallick AI. Activation of Antiviral Host Responses against Avian Influenza Virus and Remodeling of Gut Microbiota by rLAB Vector Expressing rIL-17A in Chickens. ACS Infect Dis. American Chemical Society; 2024;10:3026–41. https://doi.org/10.1021/acsinfecdis.4c00377
